# Effect of Construction Restrictions on Future Landslide Exposure Under Land-Use Simulation in Southwest China

**DOI:** 10.3390/biology15181588

**Published:** 2026-09-09

**Authors:** Jialin Jin, Wei Wang, Chutian Zhang, Cheng Wang, Xingwen Dong, Yan Yang, Jing Gao, Qingfeng Zhang

**Affiliations:** 1 College of Natural Resources and Environment, Northwest A&F University, Yangling 712100, China; 17791310617@163.com (J.J.); jaykowangwei@163.com (W.W.); chengwong@nwsuaf.edu.cn (C.W.); dxw@nwafu.edu.cn (X.D.); yyan20241015@163.com (Y.Y.); zhqf@nwsuaf.edu.cn (Q.Z.); 2 Division of immunology and Respiratory Medicine, Department of Medicine Solna, Karolinska Institutet, 17176 Stockholm, Sweden; 3 Department of Respiratory Medicine and Allergy, Center for Molecular Medicine, Karolinska University Hospital, 17176 Stockholm, Sweden

**Keywords:** CF-LR, land-use transition trajectory, ecosystem service value, spatial constraint, territorial spatial planning

## Abstract

Natural hazards significantly influence landscape patterns and ecological processes, yet their integration into land-use simulation remains limited. This study incorporates landslide susceptibility into the Patch-generating Land Use Simulation (PLUS) model to evaluate the effect of construction restrictions on future landslide exposure in Nanbu County, Sichuan Province, China. Landslide susceptibility was assessed using the certainty factor–logistic regression (CF-LR) model based on multi-period historical data, while future land-use changes (2020–2050) were projected with the PLUS model under two scenarios: a baseline scenario (without landslide constraints) and a susceptibility-constrained scenario (where high-susceptibility zones are restricted for construction). The results indicate that landslide-prone areas are spatially heterogeneous, with non-high-susceptibility zone concentrated in the west and northwest, and high-susceptibility zones clustered in the central area near the county seat. Under the baseline scenario, construction land within high-susceptibility zones increases by 107.47% over the 2020–2050 period. By contrast, the susceptibility-constrained scenario reduces construction land in these zones by 56.6% relative to the baseline 2050 projection, and by 10.01% relative to its own 2020 level. Ecosystem service value (ESV) assessment reveals that the two scenarios achieve nearly identical total ESV (with only a 0.09% difference), but the susceptibility-constrained scenario preserves 10.36 km^2^ of high-value ecosystems within high-susceptibility zones compared to the baseline scenario. These findings demonstrate that incorporating landslide susceptibility as a spatial constraint reduces future landslide exposure without compromising ecosystem service value, providing a practical, science-based framework for decision makers to inform land-use planning in mountainous regions prone to landslides.

## Simple Summary

In mountainous regions, the spatial arrangement of landscapes is crucial for maintaining ecological balance and the ability to recover from disturbances. This connection between landscape patterns and ecosystem resilience is often neglected when planning for urban growth in areas prone to natural hazards like landslides. Our study in China’s Nanbu County, Sichuan Province, addresses this gap. By combining landslide susceptibility assessment with advanced land-use simulations, we explored how future development patterns change when landslide susceptibility is considered. We found that if current trends continue, new construction will concentrate in very high-landslide-susceptibility areas, threatening both people and ecosystems. However, if landslide susceptibility is incorporated into planning, urban expansion can be redirected to safer areas while maintaining comparable overall ecosystem service value, with simulated conversion of arable land toward forest on hazardous lands. This spatially explicit approach is associated with lower disaster exposure and preserves vital ecological assets. Our findings provide a practical, science-based framework for decision makers to inform land-use planning in mountainous regions prone to landslides.

## 1. Introduction

Landslides are among the most widespread natural hazards worldwide, causing significant loss of life, infrastructure damage, and economic disruption in mountainous regions across all continents. Globally, rainfall-induced landslides affect an estimated 4.8 million people annually and cause approximately 4300 fatalities per year [1], with estimated economic losses exceeding USD 20 billion [2]. In China alone, more than 20,000 geological hazards—predominantly landslides—were recorded during 2011–2020, causing over 6000 deaths and direct economic losses exceeding CNY 63 billion [3]. Studies have shown that deforestation, urban expansion, and agricultural intensification can increase landslide frequency by two–five times [4,5] in susceptible mountainous regions, underscoring the close relationship between land-use change and landslide occurrence. Landslide occurrence is governed by multiple environmental factors, including topography (slope, aspect, and elevation), geological conditions (soil type, lithology), hydrological factors (precipitation, distance to water bodies), vegetation cover, and anthropogenic disturbance. Identifying these factors and their contributions to landslide susceptibility is the foundation for predicting current and future landslide-prone areas [6].

As urbanization accelerates, the growing population and its increasing demands are placing tremendous pressure on land resources, leading to significant shifts in urban land use. From 2015 to 2020, as urbanization accelerated, China’s urban and rural construction land area increased by 200.40 × 10^4^ hm^2^, while arable land area decreased by 65.12 × 10^4^ hm^2^, forest land area decreased by 25.25 × 10^4^ hm^2^, and grassland area decreased by 64.80 × 10^4^ hm^2^ [7]. These land-use transformations have profound impacts on regional development and ecological sustainability [8]. Land-use simulation has become a key tool for projecting these changes under alternative planning assumptions.

Conventional land-use simulation models (e.g., Markov-CA, FLUS) primarily capture the demand-driven and suitability-driven aspects of land-use change but do not incorporate landslide susceptibility as a spatial constraint. This omission means that simulated future development may encroach upon landslide-prone areas. Incorporating susceptibility as a constraint through the conversion probability layer of the spatial simulation alters where development is permitted, rather than how much total development occurs, thereby redirecting expansion away from high-susceptibility zones. However, few studies have coupled susceptibility assessment with land-use simulation in this way, leaving a critical gap in both methodology and scientific understanding [9,10,11].

Several land-use simulation models are commonly employed, including quantitative models, spatial models, and integrated models. Quantitative models primarily analyze changes in the area of different land-use types and identify the direct and indirect factors driving these changes. Spatial models, on the other hand, focus on simulating the spatial distribution of land use, often requiring the integration of quantitative data to project land-use patterns in specific years [12]. Among these, the Patch-generating Land Use Simulation (PLUS) model has been adopted in recent regional studies because its patch generation algorithm, based on multiple random seeds, captures the spontaneous emergence of new land-use patches in rapidly transforming landscapes [12]. For landslide susceptibility assessment, we selected the certainty factor–logistic regression (CF-LR) model over machine learning alternatives (Random Forest, XGBoost, SVM, and ensemble approaches). The CF module quantifies each factor class’s contribution to landslide occurrence, producing interpretable intermediate results that support planning decisions. Logistic regression also requires fewer sample points and resists overfitting when historical landslide records are limited (273 points in this study). Benchmark studies report comparable AUC values for CF-LR (0.70–0.85), Random Forest (0.75–0.88), and SVM (0.72–0.83) at regional scales, but CF-LR retains a fully transparent coefficient structure [13,14,15].

Landscape ecological responses refer to the coupled changes in ecosystem processes and hazard susceptibility that arise from modifications in the spatial configuration of land cover. When construction land expands into high-susceptibility zones, it not only increases the exposure of populations and infrastructure to landslide susceptibility, but also disrupts regulating ecosystem services such as soil retention and hydrological buffering. Conversely, restricting construction in these zones and promoting ecological restoration can simultaneously reduce hazard exposure and enhance regulating services. This dual mechanism—hazard exposure reduction and ecological service enhancement—constitutes the core of the landscape ecological response examined in this study. Ecosystem service value (ESV) was selected as the quantitative ecological response variable because it provides a monetized, comparable metric for assessing the ecological consequences of land-use decisions across different scenarios. While ESV does not encompass all dimensions of ecological health, it offers a tractable and policy-relevant indicator for comparing scenario outcomes [16,17,18,19].

Previous research has demonstrated that land-use change can alter landslide susceptibility patterns: deforestation increases slope instability by 30–50%, while afforestation and grassland restoration reduce landslide occurrence by enhancing root cohesion and reducing infiltration [20]. Studies integrating hazard assessment with land-use simulation have shown that scenario-based planning can reduce future landslide exposure by 15–40% depending on the restriction intensity [21,22,23,24].

Despite these advances, a critical gap persists at the intersection of methodological, ecological, and planning dimensions. Methodologically, few studies couple landslide susceptibility models with land-use simulation frameworks. Ecologically, the effects of susceptibility-informed land-use planning on future landscape dynamics and ecosystem services remain unknown. From a planning perspective, susceptibility-informed land-use scenarios that restrict construction in high-susceptibility zones have not been systematically evaluated. This study addresses all three dimensions simultaneously by posing the following central question: Can incorporating landslide susceptibility as a spatial constraint in land-use simulation effectively reduce future landslide exposure while maintaining ecosystem service value?

We hypothesize that restricting construction within high-susceptibility zones will (1) substantially reduce future construction land exposure to landslide-prone areas relative to uncontrolled development. The hypothesis is formulated directionally rather than with a specific quantitative threshold, because no planning criterion or previous study provides an independent a priori basis for a particular percentage reduction in this setting. Here, landslide exposure is operationalized as the area of construction land located within high-susceptibility zones, which serves as a spatial proxy for the population and assets exposed to landslide hazard; (2) maintain comparable total ecosystem service value within hazardous zones. The baseline scenario serves as the control, whereas the susceptibility-constrained scenario represents the treatment in this natural experimental framework. Future land-use changes are projected based on the continuation of historical trends as captured by the Markov chain module, which derives transition probabilities from observed land-use trajectories. These transitions are driven by multiple factors including urbanization pressure, agricultural restructuring, ecological restoration policies (e.g., the Grain-to-Green program) [20], and infrastructure expansion. The PLUS model captures the spatial relationships between these driving factors and land-use changes, projecting their future spatial distribution under the assumption that current socioeconomic and policy trends continue.

The mountainous region of Southwest China is particularly suitable for this study because it exhibits high landslide frequency driven by complex terrain, concentrated summer rainfall, and intensive human disturbance. Sichuan Province alone recorded over 8000 geological hazards during 2011–2020, with Nanbu County experiencing recurring landslide events [25] that have caused infrastructure damage and economic losses. This study aims to (1) assess landslide susceptibility using the CF-LR model; (2) simulate future land-use changes (2020–2050) under a baseline scenario and a susceptibility-constrained scenario using the PLUS model; and (3) conduct complementary analyses to compare the two scenarios in terms of landslide exposure, land-use transition pathways, and ESV. This integrated approach allows us to determine whether susceptibility-informed planning can achieve disaster mitigation without compromising ecological benefits, offering insights for regional urban development and disaster prevention.

## 2. Materials and Methods

### 2.1. Study Area

**Figure 1:**
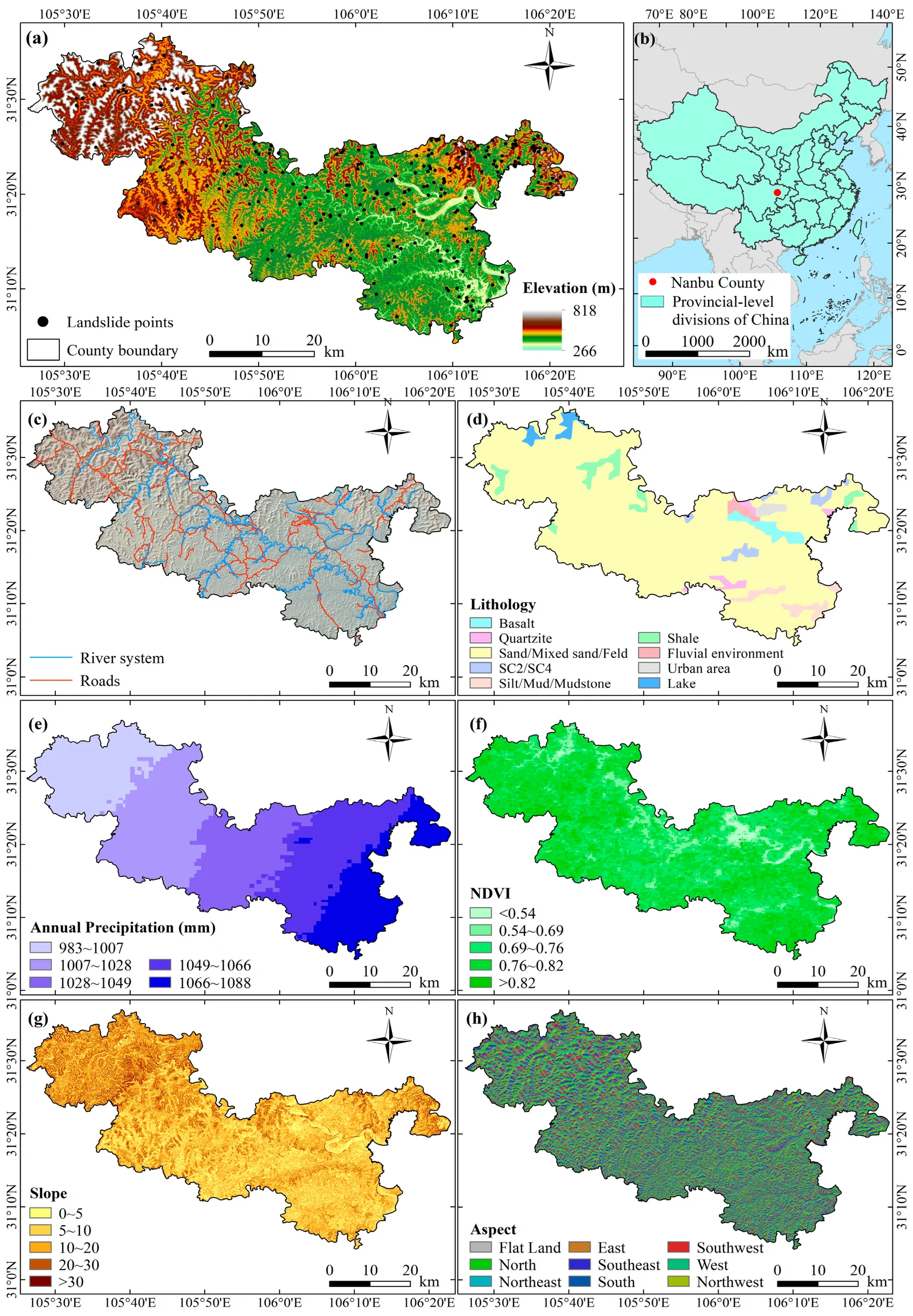
Overview of the study area and environmental factors. (**a**) Spatial distribution of historical landslide points and elevation of Nanbu County; (**b**) location of Nanbu County in China; (**c**) river system and roads; (**d**) surface lithology; (**e**) annual precipitation; (**f**) normalized difference vegetation index (NDVI); (**g**) slope; and (**h**) aspect. Data are compiled from public datasets, see Table 1.

Nanbu County is situated in the northern part of the Sichuan Basin and the northwest of Nanchong City, between 105°27′~106°24′ E, 31°4′~31°30′ N, with an area of 2212 km^2^. The county features undulating hills, with the terrain rising to the northwest and sloping down to the southeast. The topography is predominantly dendritic due to the influence of the river systems, and it is characterized by inland lacustrine sedimentary clastic rock formations, which are geologically unstable. The distribution of historical landslide disaster points in Nanbu County is shown in Figure 1.

Surface water erosion and infiltration significantly reduce the stability of the soil mass. Nanbu County experiences a mid-subtropical humid monsoon climate, with highly uneven rainfall distribution; it is more abundant in the southeast and less in the northwest. The region experiences a synchronous occurrence of heat and rainfall, making it prone to landslides and other geological hazards during the concentrated summer precipitation periods [25].

### 2.2. Data Acquisition

The process of selecting potential landslide susceptibility factors for a region involves conducting a detailed landslide investigation to understand the link between the regional geological environment prone to failures and the distribution of landslide occurrences. This involves analyzing the specific characteristics and the mechanistic causes of landslide occurrences, then choosing those factors that significantly control or influence landslide behavior and assigning appropriate intervals or value ranges [26]. In the selection of evaluation factors, besides relying on prior expertise from similar studies, one needs to take into account the local realities of the research area and the availability of data. This usually involves weighing the influence of each factor. It is also important to account for correlations between factors; highly correlated ones may be pared down to maintain simplicity and minimize redundancy in the analysis.

**Table 1:** Summary of datasets used in this study.

Data Name	Spatial Resolution	Landslide Susceptibility Assessment	Land-Use Simulation	Data Source
Historical Landslide Points Data	-	√	-	[27]
2010, 2015, 2020 Land-Use Data	30 m	-	√	[28]
Elevation	30 m	√	√	[29]
Slope	30 m	√	√	Derived from DEM
Aspect	30 m	√	√	Derived from DEM
Soil Type	1 km	√	√	[30]
GDP	1 km	-	√	[31]
Population	100 m	-	√	[32]
Annual Precipitation	~1 km	√	√	[33]
Annual Average Temperature	~1 km	√	√	[33]
Annual Average NDVI	30 m	√	-	[34]
Surface Lithology	-	√	-	[35]
Distance to Road	-	√	√	[36]
Distance to Watercourse	-	√	√	[36]

Note: Elevation, slope, and aspect were derived from the Digital Elevation Model (DEM); GDP and NDVI refer to Gross Domestic Product and Normalized Difference Vegetation Index, respectively. “√” indicates that the data are utilized in the corresponding analysis procedures; “-” refers to not applicable.

Twelve environmental variables were selected based on their documented influence on landslide occurrence and land-use change: elevation, slope, and aspect capture topographic controls; soil type and surface lithology represent geological conditions; annual precipitation and temperature reflect climatic drivers; NDVI indicates vegetation cover; GDP and population represent socioeconomic pressure; and distance to road and watercourse are proxies for accessibility and hydrological disturbance. The historical landslide disaster points data came from the Geographic Remote Sensing Ecological Network; the data of Nanbu County are extracted and imported into ArcGIS (v10.7); and the projection is set as the WGS_1984_UTM_Zone_48N spatial coordinates. The slope and aspect data for Nanbu County were derived from the Digital Elevation Model with a resolution of 30 m using the slope and aspect tools in ArcGIS. The distances to road networks and water systems were calculated using the Euclidean distance tool. Other data sources are shown in Table 1.

All raster datasets were projected to the WGS_1984_UTM_Zone_48N coordinate system and resampled to 30 m. Categorical data (soil type at 1 km) were resampled using nearest-neighbor assignment; continuous variables (precipitation, temperature) used bilinear interpolation. All layers were clipped to the Nanbu County boundary and aligned to a common grid. We selected 30 m to match the land-use data resolution in the PLUS model and to align with conventions in comparable regional-scale susceptibility studies. This resolution is also consistent with the soil database from the Chinese Academy of Sciences Resource and Environmental Science Data Center, the most accurate soil database available for regional-scale work in China. Resampling 1 km source data to 30 m does not create new information, but this approach is standard practice in regional landslide susceptibility assessment [6]. We therefore distinguish the computational grid resolution from the native information content of each predictor: the effective spatial resolution of the climatic and soil layers remains 1 km, and the 30 m grid represents a common computational geometry rather than finer-grained environmental information. The resulting susceptibility surface is accordingly smooth over these coarse-resolution inputs and cannot resolve small-scale variation controlled by sub-kilometer climatic or edaphic gradients; this spatial pseudoprecision is considered when interpreting the susceptibility maps.

### 2.3. Methodology

**Figure 2:**
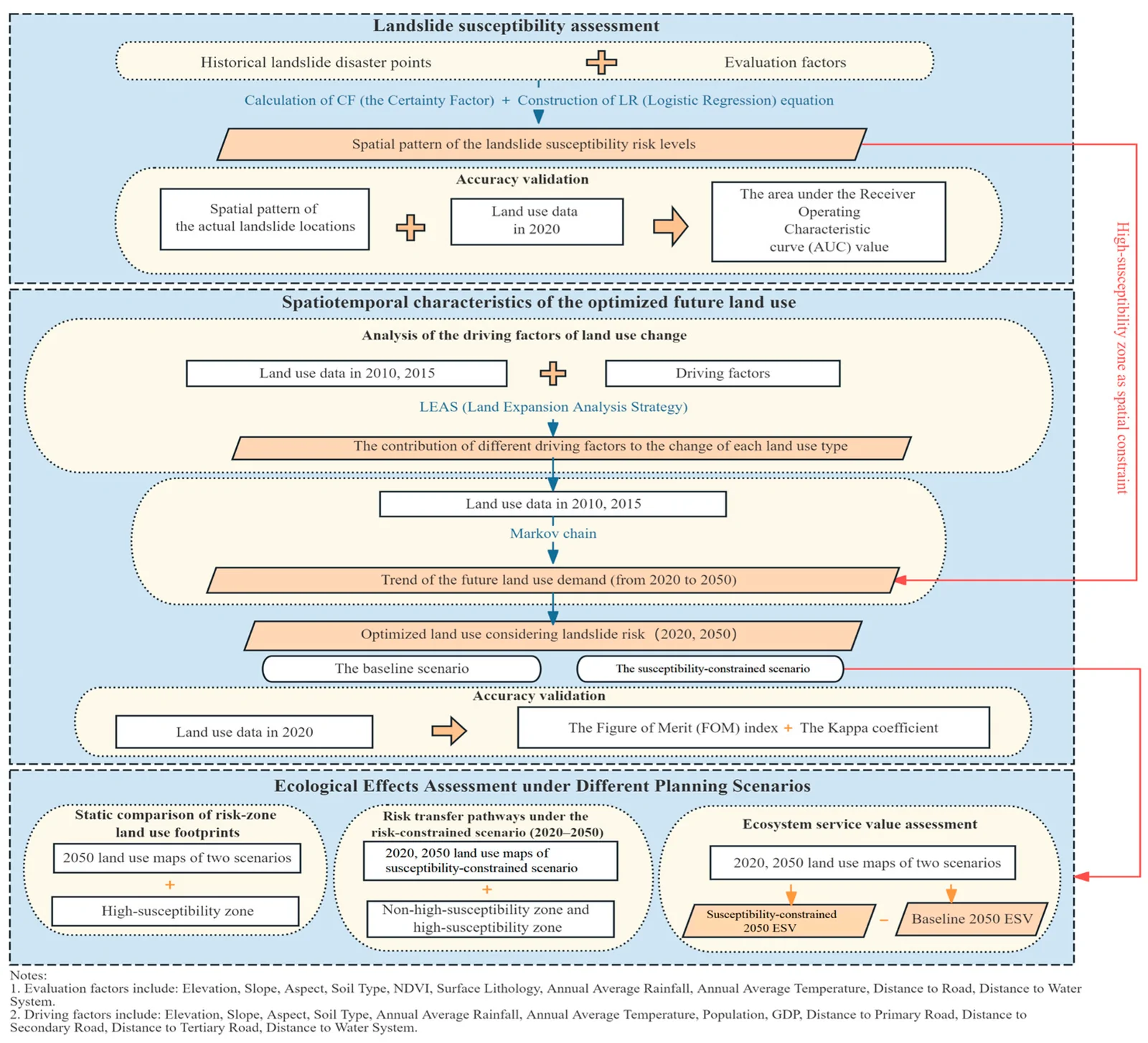
Technical route.

The model was trained to capture the change dynamics or processes of various land uses based on the land-use data of Nanbu County for the years 2010 and 2015; then, the model was used to project 2020 outcomes based on that process. The accuracy would be validated by comparing the simulated 2020 results to the actual 2020 figures. This study evaluates the landslide susceptibility of Nanbu County from the perspective of landslide susceptibility, simulates future land-use changes, and compares the results with those observed. (1) Using the CF-LR model, the mechanism of landslide occurrence is calculated, and the certainty factors of ten influencing factors including elevation, slope, aspect, soil type, normalized difference vegetation index, surface lithology, annual precipitation, annual average temperature, distance to roads, and distance to watercourses are selected. A logistic regression model for landslide occurrence within Nanbu County is established, ultimately yielding the spatial distribution results of landslide susceptibility in Nanbu County. (2) Three periods of land-use data from 2010, 2015, and 2020, along with twelve influencing factors including elevation, are selected. The parameters of the CA based on the Multiple Random Seeds (CARS) module in the PLUS model are adjusted. Starting from the land-use data of 2015, the land-use pattern of 2020 is simulated. The accuracy is verified through Kappa coefficients and Figure of Merit (FOM) coefficients. (3) Through the Markov chain module in the PLUS model, starting from 2020, the land-use demand quantities for every five years between 2020 and 2050 are simulated. Additionally, the results of landslide susceptibility assessment are considered in the future land-use simulation, and the differences between the two scenarios are compared and analyzed. The technical route for this study is shown in Figure 2:

#### 2.3.1. CF-LR Model

The certainty factor (CF) is a probabilistic indicator based on the statistical relationship between known landslide hazard points and influencing factors, used to analyze the sensitivity of each evaluation factor affecting landslide occurrence [37]. The formula for the certainty factor is as follows:

(1) $$CF=\left\{\;\begin{array}{c} \frac{PP_{a}-PP_{s}}{PP_{a}(1-PP_{s})},\;PP_{a}\geq PP_{s}\\ \frac{PP_{a}-PP_{s}}{PP_{s}(1-PP_{a})},PP_{a}<PP_{s} \end{array}\right.$$

In the formula, *PP_a_* represents the probability of landslide occurrence within the evaluation factor a, which is the ratio of the number of landslide points in a specific level range of factor a to the area corresponding to that range; *PP_s_* represents the prior probability of landslide occurrence across the entire study area, which is the ratio of the total number of landslide points to the area of the study area [38,39]. The range of CF values is [−1, 1], where a CF value greater than 0 (CF > 0) indicates a higher likelihood of landslide occurrence, with values closer to 1 indicating a higher probability; a CF value less than 0 (CF < 0) indicates a lower likelihood of landslide occurrence, with values closer to −1 indicating a lower probability; and a CF value of 0 (CF = 0) indicates uncertainty in the probability of landslide occurrence [40].

The logistic regression (LR) model is a multivariate statistical analysis method for studying binary dependent variables. It can describe various complex nonlinear relationships between natural phenomena through simple linear regression [41]. The formula for the logistic regression model is as follows:

(2) $$\left\{\;\begin{array}{c} Z=\alpha +\beta_{1}x_{1}+\beta_{2}x_{2}+\cdots +\beta_{n}x_{n}\\ P=\frac{e^{Z}}{1+e^{Z}} \end{array}\right.$$

In the formula, *P* represents the probability of a landslide occurring, with a range of [0, 1]. For the sample points, the binary dependent variable is coded as 1 for landslide presence and 0 for landslide absence; *Z* represents the linear predictor (i.e., the log-odds of landslide occurrence), *x*_1_, *x*_2_, …, *x*_n_ are the CF values of each classification level in the respective factors; $\beta_{1}$, $\beta_{2}$,… $\beta_{n}$ are the logistic regression coefficients, α is the model intercept.

In general, the CF is a method used to assess the susceptibility of landslides. Its value, being positive, negative, or in between, indicates the likelihood of a slope failing. A positive CF implies an increased likelihood of a slide happening, while a negative suggests less tendency. The absolute magnitude signifies the severity or extent of the potential collapse. Before the model calculation, based on the CF extracted to the sample points, multiple collinearity checks were performed using IBM SPSS Statistics 27. The tolerance (TOL) of the selected factors is all greater than 0.1, and the variance inflation factor (VIF) is less than 10, indicating that there is no collinearity problem between the factors, which can be used to establish the logistic regression model. The CF values calculated for each type were extracted to each sample point, and logistic regression modeling was carried out using SPSS 27.

The certainty factor–logistic regression (CF-LR) model integrates the CF values of different grades of each evaluation factor calculated by the CF model as independent variables in binary logistic regression analysis. It obtains the regression coefficients for each evaluation factor and establishes the corresponding logistic regression equation, ultimately conducting an assessment of landslide susceptibility [42]. In the landslide susceptibility assessment, non-landslide sample points were selected from outside the buffer zone. Generally, there is a risk of recurrence within a certain range near historical landslide disaster points, so the distance to landslide points should be considered when selecting non-landslide points. Li et al. introduced disaster point buffer zones in their study of land-use response to geological disasters, exploring response mechanisms within the zone [43]. The determination of the optimal buffer distance in research is related to the environmental characteristics and data sources of the selected study area, and it requires repeated experiments for better determination [44]. Similarly, this study randomly selects non-landslide points outside the buffer zone and considers the landslide susceptibility levels of different landslide points, ultimately setting a 500 m buffer distance. The impact of buffer zones does not affect the selection of non-sliding points, which are placed at a certain distance away from the sliding points to ensure representativeness. This is carried out by using a random point generation tool in ArcGIS, where without a distance restriction some non-sliding points may end up closer to the slides. Since landslide areas are the minority and non-landslide areas are the majority, increasing the ratio of non-landslide areas helps the model better reflect the real relationship between landslides and non-landslides within the study area [45]. Based on the experience of other studies [46], 819 non-historical landslide points were randomly selected outside the area at a ratio of 1:3 using the random point generation tool of ArcGIS, forming a total of 1092 sample points required for the model.

#### 2.3.2. PLUS Model

The PLUS model is a raster-based patch-generating land-use simulation model that simulates the generation of new land-use patches by capturing the relationship between land-use type changes and the driving factors influencing these changes. The PLUS model consists of two modules: the Land Expansion Analysis Strategy (LEAS) and the CARS model. The LEAS module uses a Random Forest algorithm to fit the probability of change of each land-use type, and outputs both the development probability of each land-use type and the variable importance of the driving factors [47]. The CARS module is a CA model that introduces a growth mechanism based on multiple random patch seeds. It combines random seed generation and a threshold decrement mechanism to dynamically simulate patch generation at a micro level, thereby achieving spatial visualization of land-use changes [48].

For land-use simulation purposes, the very high-susceptibility class was aggregated into a high-susceptibility zone, while the remaining four classes were combined into a non-high-susceptibility zone. This binary zonation served as the spatial constraint layer in the CARS module. In the baseline scenario, no landslide susceptibility constraints were applied, and land-use transitions followed historical trends. In the susceptibility-constrained scenario, we imposed a constraint on land-use conversion to construction land in high- and very high-susceptibility zones.

Using land-use data from 2010 and 2015, along with 12 influencing factors such as elevation, the parameters of the PLUS model were calibrated. The probability for random patch seeds (Pgt) was set at 0.9, the decay coefficient (Ec) for the decay threshold was set at 0.1, neighborhood range (NS) was set to 3, the policy intensity (Dw) targeting construction land-use types was set at 0.5, and the maximum proportion of random seeds (PoS) was set at 0.0001. Finally, the calibrated PLUS model was applied to simulate the land use in 2020, which was then compared and validated against observed 2020 data. To evaluate the sensitivity of the simulation to parameter choices, a one-factor-at-a-time (OAT) test was conducted: each of the five parameters (Pgt, Ec, NS, Dw, and PoS) was independently varied across four alternative values (20 iterations), and the resulting 2020 maps were compared with the susceptibility-constrained simulated 2020 map using the Figure of Merit (FOM; see Section 2.3.3). For the 2050 projection, because no reference map exists for 2050, long-term stability was instead assessed by the Kappa coefficient between each perturbed 2050 simulation and the susceptibility-constrained 2050 simulation. The PLUS model was implemented in PLUS v1.3.5 (https://github.com/HPSCIL/Patch-generating_Land_Use_Simulation_Model) (accessed on 20 October 2022).

#### 2.3.3. Accuracy Validation

The results of the landslide susceptibility assessment are validated using the Receiver Operating Characteristic (ROC) curve. The closer the ROC curve is to the upper left corner, the higher the model’s accuracy. Typically, the area under the curve (AUC) value is used to represent the accuracy of the prediction results, with a range generally between [0.5, 1]. A value closer to 1 indicates that the research findings are more accurate [49].

The results of the land-use simulation are verified using the Kappa coefficient and the FOM index. The Kappa coefficient is often used to describe the changes in raster datasets over time and the direction of change, as well as to test the correctness of image classification for real-world object judgments. The FOM provides a quantitative validation at the cell scale: the larger its value is, the higher the accuracy of the results used for land-use simulation will be, with a range generally between [0.01, 0.25] [50]. The calculation formula is as follows:

(3) $$FOM=\frac{A}{A+B+C+D}$$

In the formula, *A* represents the part that has changed but was incorrectly changed, *B* represents the part that has changed and was correctly changed, *C* represents the part that was predicted to change but did not actually change, and *D* represents the part that was predicted not to change but did actually change.

AUC was calculated in SPSS 27, the Kappa coefficient and FOM index were inherently implemented in the PLUS model (v 1.3.5).

#### 2.3.4. Ecosystem Service Value (ESV) Assessment

To assess the ecological consequences of the simulated land-use scenarios, we conducted an ecosystem service value (ESV) assessment using the equivalent factor method [16]. The standard equivalent value D was calculated based on Nanbu County’s statistical yearbook [51] for 2020 and Equation (4):

(4) $$D=\frac{1}{7}\times \left(S_{r}\times P_{r}+S_{w}\times P_{w}+S_{c}\times P_{c}\right)$$

where D is the value of one equivalent factor (CNY/ha); $S_{r}$, $S_{w}$, and $S_{c}$ are the proportions of sown area of rice, wheat, and corn to the total sown area of these three crops (%); and $P_{r}$, $P_{w}$, and $P_{c}$ are the value of yield per hectare of rice, wheat, and corn (CNY/ha), see Table S1 in Supplementary Material (SI).

ESV coefficients for arable land, forest land, grassland, water body, and construction land were derived from the standard equivalent factor table [16]. For arable land, the coefficients were weighted by the proportion of dryland (70%) and paddy field (30%) in Nanbu County [52]. For forest land, coefficients for coniferous forest were applied, as over 90% of the county’s forest area is coniferous [53].

We then derived the ecosystem service equivalent values per unit area for each land-use type (Table S2). These equivalent values were multiplied by D to obtain the ecosystem service value per unit area for each land-use type in Nanbu County (Table S3). Applying these coefficients, we calculated the total ESV via Microsoft Excel 2016 and its spatial distribution for 2020 and for the two 2050 scenarios through raster calculations in ArcGIS 10.7.

Total ESV and its spatial distribution were calculated for 2020 and for the 2050 baseline and susceptibility-constrained scenarios using raster calculations in ArcGIS. An ESV gain–loss map was generated by subtracting the baseline 2050 ESV from the susceptibility-constrained 2050 ESV to identify areas where susceptibility-constrained planning preserves higher ecological value.

## 3. Results

### 3.1. Landslide Susceptibility Assessment

#### 3.1.1. Varying Landslide Susceptibility Across Different Evaluation Factor Ranges

Based on the selected evaluation factors, the certainty factor (CF) values for different levels of each evaluation factor were calculated using the CF model, and the results are shown in Table S4. Evaluation factors such as elevation, slope, NDVI, and annual precipitation, etc., are classified using the natural breaks method in ArcGIS.

From Table S4, it can be seen that the area with an elevation between 372 and 422 m is more prone to landslide disasters compared to other grading ranges. The slope with an angle between 20° and 30° is also more prone to landslide disasters compared to other grading ranges. In terms of aspect, the west and southwest directions are more susceptible to landslide disasters.

In terms of soil types, the newly deposited soil and yellow soil categories are more susceptible to landslide disasters. Although the purple soil category has a historical number of landslide points reaching 204, due to its area of 1690.43 km^2^, which is much larger than other soil types, the certainty factor is relatively lower. A similar situation also exists for the factor of surface lithology.

Han et al. analyzed the impact of land use on landslides. Due to the anchoring of vegetation roots in forested and grassy areas and the reduced infiltration of rainfall, under the same geological and geomorphological conditions, regions with higher vegetation coverage have a lower probability of landslides compared to areas with lower vegetation coverage [54]. However, from the perspective of NDVI in this study, it is not necessarily true that the more vegetation cover, the less prone a place is to landslide disasters, because the study area is characterized by large areas of farmland, where artificial vegetation has a more monotonous composition compared to natural vegetation, resulting in poorer surface stability. In local regions, even areas with higher vegetation coverage may still experience landslide disasters.

Areas with higher rainfall and higher temperatures are more likely to have landslide disasters. Under high-temperature conditions, the surface soil layer cracks, reducing its stability, and landslide disasters are more likely to occur during the concentrated rainfall in summer.

The certainty factor is relatively high for areas within 700 m of the road and beyond 3800 m, indicating that the proximity to roads, influenced by human activities, is more prone to landslide disasters. In areas far from roads, in addition to the distance from roads, it is also affected by other factors. Areas within 900 m of the water system are more prone to landslides, as the closer to the river, the more susceptible to surface runoff and groundwater erosion, leading to insufficient surface stability.

To provide a concise, class-resolved overview, the factor classes most strongly associated with each of the five susceptibility levels are consolidated in Table 2. Specifically, the very high-susceptibility class is dominated by urban lithology (CF = 0.676), basalt (CF = 0.589), newly deposited soil (CF = 0.546), and yellow soil (CF = 0.537), together with high annual precipitation (1049–1066 mm, CF = 0.453). By contrast, the very low-susceptibility class is characterized by high elevation (566–818 m, CF = −0.453), lake areas (CF = −0.717), and locations far from the water system (>4900 m, CF = −0.749).

#### 3.1.2. Quantitative Relationship Between Landslide Occurrence and Evaluation Factors

The complete fitting results for all independent variables in the CF-LR model are presented in Table S5. Among them, the *p*-values for elevation, soil type, and annual average temperature are greater than 0.05 and are not statistically significant, thus they need to be excluded. The final regression equation obtained regarding the probability of landslide occurrence is as follows:

(5) $$\left\{\begin{array}{c} \begin{array}{c} Z=-1.094+1.518x_{1}+1.162x_{2}+1.198x_{3}+1.216x_{4}+1.586x_{5}+1.202x_{6}+1.218x_{7}\\ P=\frac{e^{Z}}{1+e^{Z}} \end{array} \end{array}\right.$$

In the equation, the variables $x_{1}$ to $x_{7}$ represent the corresponding CF (certainty factor) values for each grading range of slope, slope aspect, NDVI, surface lithology, annual precipitation, distance to road, and distance to water system, respectively. In the specific computation, it quantifies the combined impact of various factors on that grid. All seven regression coefficients are positive. Because the predictors entering the LR model are CF-transformed factor classes rather than the original environmental variables, a positive coefficient indicates that factor classes with higher CF values, that is, classes more strongly associated with observed landslides, receive higher predicted susceptibility; it does not imply that increasing the original variable increases landslide susceptibility. For factors whose classes exhibit both positive and negative CF values, such as NDVI and distance to road (Table S4), the sign of the class-level CF values does not transfer to the underlying continuous gradient, and the ecological direction of the original variable must instead be read from the pattern of class-level CF values. The sensitivity of the study area to landslides, as inferred from the regression coefficients of the evaluation indicators, is as follows: annual average precipitation, slope, distance to water system, surface lithology, distance to road, NDVI, and aspect, in descending order; this ranking reflects the relative contribution of each CF-transformed predictor to the fitted log-odds rather than the direction or magnitude of the effect of the original environmental gradient.

#### 3.1.3. Spatial Pattern of the Landslide Susceptibility Levels

The CF-LR model showed moderate predictive power, with an AUC of 0.708 from the ROC curve. We also assessed the spatial autocorrelation of the CF-LR residuals to verify the assumption of spatial independence. The global Moran’s I indicates weak to moderate spatial autocorrelation (0.137–0.220 across four spatial weight specifications; e.g., 0.144 under K-nearest-neighbor weights, z = 10.35, *p* < 0.001; Table S6), and the empirical semivariogram reaches its sill at a short range of ~2.2 km, indicating moderate, strongly localized residual dependence (Figure S1). To evaluate whether this short-range residual dependence affects predictive performance, we performed spatially blocked cross-validation (leave-one-block-out with spatially contiguous blocks, refitting the logistic regression within each fold). The pooled AUC decreased from 0.708 to 0.652–0.680, indicating that residual spatial autocorrelation inflates the apparent AUC by approximately 0.028–0.056 while the model retains substantial discriminative ability (Table S7). Residual spatial dependence is therefore treated as a limitation of the CF-LR model.

**Figure 3:**
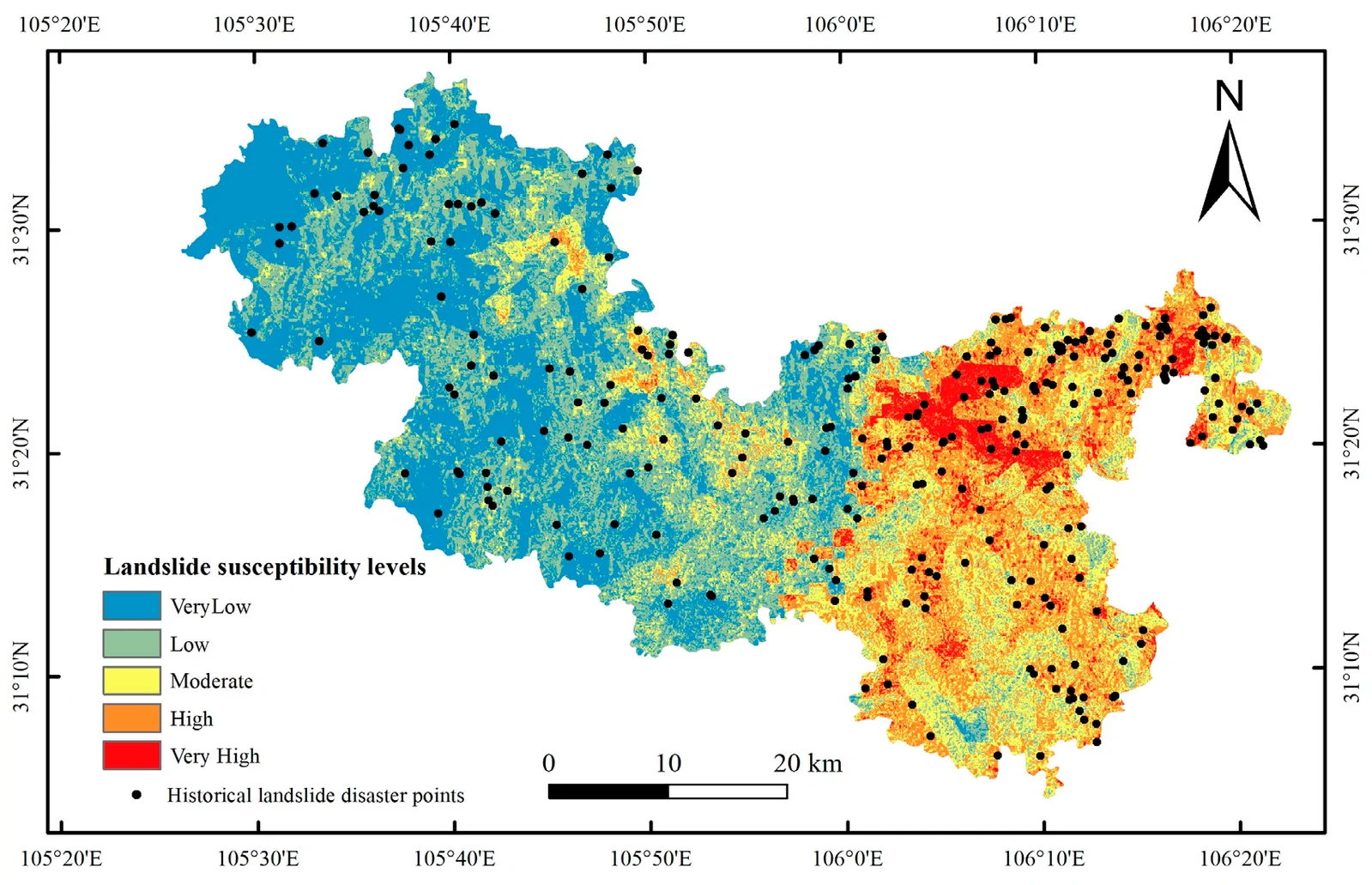
Landslide susceptibility levels map in Nanbu County.

Based on the CF-LR model’s probability regression equation, we conducted raster calculations to generate the landslide susceptibility probability distribution for Nanbu County (Figure S2). Using the natural breaks method, these probability values were then classified into five susceptibility levels: very low (<0.142), low (0.142–0.244), moderate (0.244–0.367), high (0.367–0.516), and very high (≥0.516). The resulting susceptibility map is shown in Figure 3. From the map, it is evident that the areas with very low susceptibility to landslides are predominantly located in the western and northwestern parts of Nanbu County, while the areas with very high susceptibility are mainly in the central part, particularly concentrated around the county town.

### 3.2. Spatial Optimization of Future Land Use

#### 3.2.1. Validation and Sensitivity Analysis of the Land-Use Simulation

**Figure 4:**
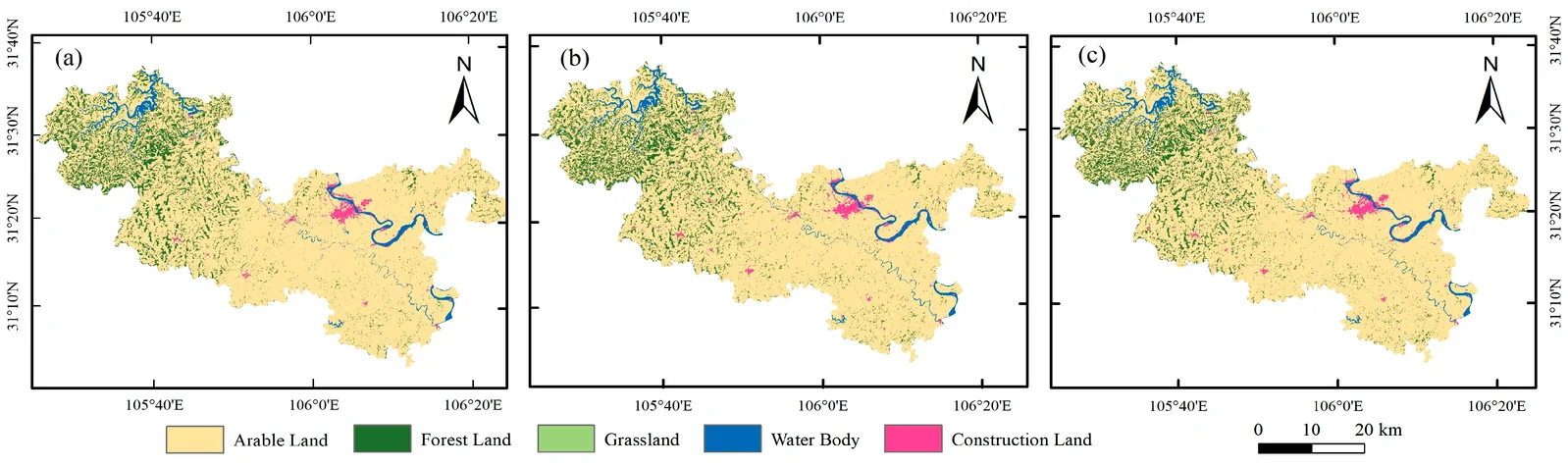
Land use under the actual situation (**a**), the susceptibility-constrained scenario (**b**), and the baseline scenario (**c**) in Nanbu County in 2020.

The spatial distribution of land use in Nanbu County in 2020 under the actual situation, the susceptibility-constrained scenario, and the baseline scenario is shown in Figure 4. In the susceptibility-constrained scenario, the Kappa coefficient is 0.793, the overall accuracy is 0.939, and the FOM is 0.0991. Among them, the accuracies of arable land and water areas are relatively high, 0.963 and 0.967, respectively, while the accuracies of construction land, forest land, and grassland are 0.835, 0.781, and 0.714 respectively. In the baseline scenario, the Kappa coefficient is 0.791, the overall accuracy is 0.938, and the FOM is 0.0991. Specifically, the accuracies of arable land and water areas are relatively high, reaching 0.963 and 0.968 respectively, and the accuracies of construction land, forest land, and grassland are 0.816, 0.778, and 0.556 respectively, indicating moderate accuracy of results in both scenarios.

In the OAT test, the FOM remained within ±1.5% of the reference value (i.e., 0.0991 obtained from the susceptibility-constrained 2020 simulation) across all perturbations of the four CARS parameters (probability for random patch seeds, decay coefficient, maximum proportion of random seeds, and policy intensity). The neighborhood range is the exception: increasing NS beyond 3 degraded the FOM by 27.5–38.1% (Table S8), so the simulation is not insensitive to all parameters. Larger values of NS impose stricter neighborhood competition and suppress the expansion of changed land-use patches, which underallocates changed cells and lowers the FOM. The calibrated value NS = 3 was therefore retained, but the reported FOM is conditional on this setting, and the sensitivity to NS should be re-evaluated whenever the framework is transferred to another region. Nevertheless, the spatial patterns of the susceptibility-constrained simulated 2020 maps showed little variation across the 20 iterations (Figure S3).

Derived from the Random Forest within the LEAS module, variable importance for changes in different land-use types is assessed (Table 3), larger values indicate a stronger contribution of that factor to the predicted change of the corresponding land-use type. Specifically, annual precipitation has the strongest impact on the development of arable land and forest, while grassland is most affected by GDP. Water body is strongly influenced by elevation. Construction land is mainly driven by distance to tertiary road, population, and GDP.

#### 3.2.2. Trend of Future Land-Use Demand

The Markov chain stochastic model can simulate and predict the development trend of land through the transition probability matrix, on the basis of the occurrence probability of multi-period land types. Choosing land-use data from the years 2015 and 2020, considering that a Markov chain requires information from the previous time step for the next, we estimate the land-use demands over the period 2025 to 2050 through prediction intervals of five-year intervals, using the chain to move forward from 2020. The results are shown in Table 4: it can be seen that from 2025 to 2050, the number of arable land units in Nanbu County gradually decreases, while the number of forest land and construction land units shows an increasing trend. The number of grassland and water body units remains relatively stable.

#### 3.2.3. Spatiotemporal Characteristics of the Optimized Future Land Use

Spatially, arable land in Nanbu County is predominantly located in the central and eastern regions, while forest lands are mainly in the northwest. Urban construction lands are distributed along the Jialing River, and town construction lands are situated near roads and rivers. Under the baseline scenario, there is a clear spatial clustering effect of construction land near urban areas, with construction land along Binjiang Street developing towards the northwest and southeast along the river. Construction lands in the central and southwestern parts along the river in Hedong Town are gradually developing and merging. There is also noticeable expansion of construction lands in Nanlong Town, Dingshui Town, Dongba Town, Jianxing Town, and Fuhu Town. In contrast, under the susceptibility-constrained scenario, the expansion of construction land on Binjiang Street avoids the designated high-landslide-susceptibility zones and the expansion rate is noticeably slower. Furthermore, as can be seen from Table S9 and Figure S4, the Kappa coefficient between each perturbed prediction and the susceptibility-constrained 2050 simulation ranged from 0.812 to 0.989, indicating that the projected spatial pattern is highly stable to parameter perturbation.

**Figure 5:**
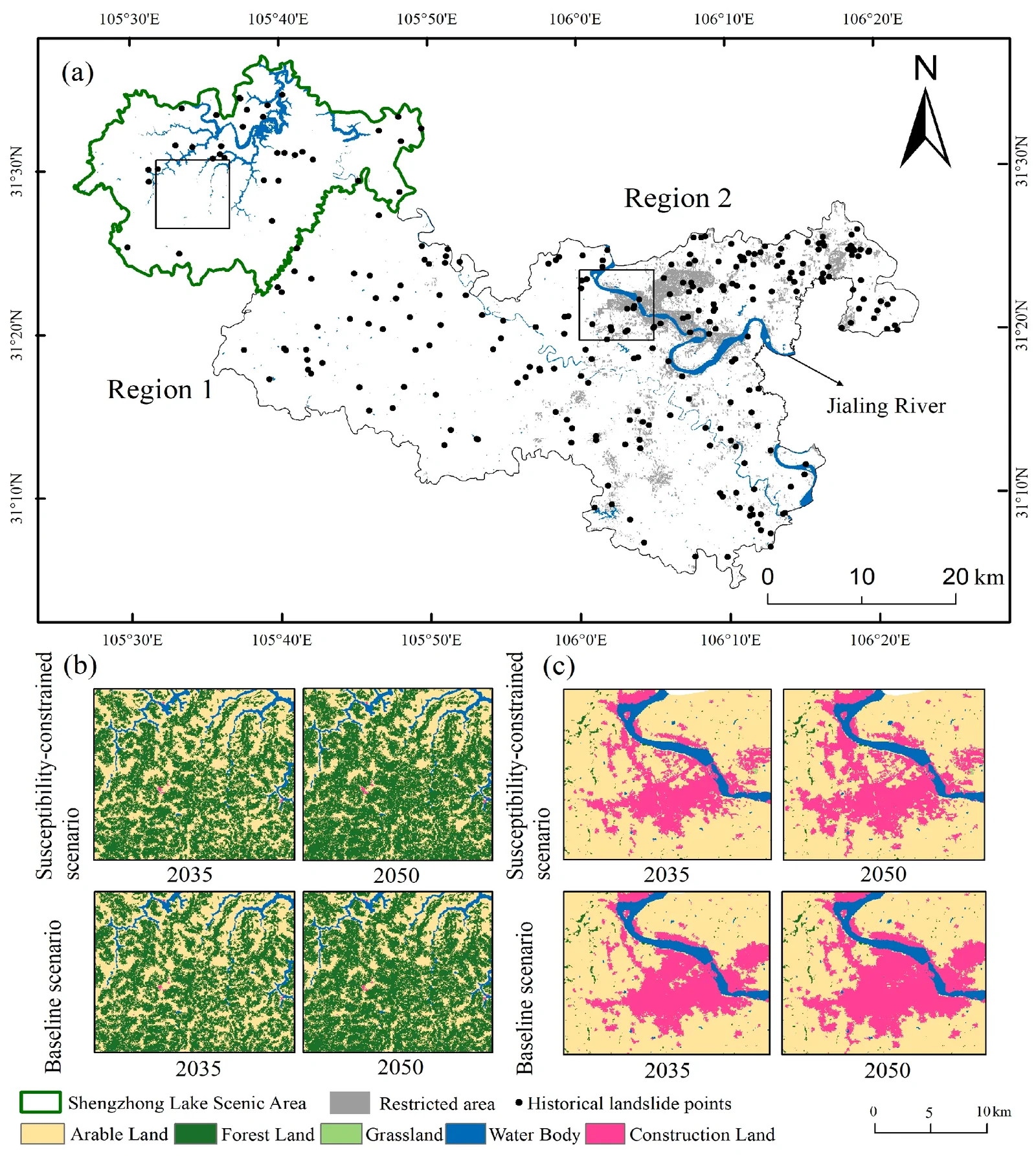
(**a**) Spatial optimization of future land use; (**b**) land-use pattern in Region 1; and (**c**) land-use pattern in Region 2.

To analyze the change characteristics of land-use patterns under different scenarios, two regions with significant changes were selected for an in-depth study (Figure 5). In Region 1, located in the northwest, there was originally extensive forest land distribution with few historical landslides; thus, this area is unrestricted and shows no significant difference between the two scenarios. Over time, other arable lands within Region 1 are also gradually converted to forest land, which may contribute to lower susceptibility probability in the model. From the perspective of local development, this region’s strategy to create a wetland park around Shengzhong Lake and strengthen ecological protection around the reservoir aligns with these changes. In both scenarios, the construction land in Region 2 near the urban area gradually expands over the years. The difference is that under the susceptibility-constrained scenario, the construction land tends to expand outward, avoiding high-susceptibility zones. Under the baseline scenario, the construction land shows a clustering pattern, forming large contiguous areas that cover the restricted areas in the susceptibility-constrained scenario. In this scenario, large-scale urban expansion poses certain risks.

### 3.3. Ecological Effect Assessment Under Different Planning Scenarios

#### 3.3.1. Static Comparison of Susceptibility Zone Land-Use Footprints

We overlaid the 2050 land-use maps of both scenarios with the high-landslide-susceptibility zone to quantify land-use dynamics within and outside hazardous areas (Table 5). Within the high-susceptibility zone, the baseline scenario exhibited a 107.47% increase in construction land (from 8.52 km^2^ to 17.69 km^2^), alongside a 47.55% increase in forest land, a 76.41% decrease in grassland, a 12.17% decrease in arable land, and a 4.47% increase in water body. This pattern suggests a passive transition where arable land abandonment simultaneously feeds both ecological succession and construction encroachment, while the increase in water body area likely reflects the expansion of existing water bodies into adjacent arable land through natural inundation or irrigation infrastructure development. Under the susceptibility-constrained scenario, construction land within the high-susceptibility zone decreased by 10.01% (from 8.52 km^2^ to 7.67 km^2^), while forest land increased by 20.61%, grassland increased by 130.61% (from a very small base of 0.07 km^2^ to 0.16 km^2^), and water body (−0.08%) as well as arable land remained nearly stable (+0.42%). This pattern is consistent with a planning strategy that restricts development, preserves agricultural land, and facilitates conversion toward forest and grassland in hazardous areas, with the notable grassland expansion reflecting potential revegetation on abandoned lands. Within the non-high-susceptibility zone, the susceptibility-constrained scenario absorbed development pressure diverted from high-susceptibility areas, resulting in a 154.53% increase in construction land (from 21.22 km^2^ to 54.01 km^2^), which is substantially higher than the 106.63% increase under the baseline scenario (from 21.22 km^2^ to 43.85 km^2^). Both scenarios achieved similar forest land gains in non-high-susceptibility zone (approximately 56% increase). Water body in the non-high-susceptibility zone increased modestly from 57.22 km^2^ in 2020 to approximately 60 km^2^ by 2050 (approximately 5% increase). This moderate expansion reflects the continued presence and slight enlargement of existing water bodies (e.g., Shengzhong Reservoir), rather than a dramatic landscape transformation. Critically, total forest land across the entire study region increased substantially under both scenarios, while the non-high-susceptibility zone already contained extensive water bodies in 2020 that remained largely stable, with only minor increases.

Notably, the 10.02 km^2^ difference between the two 2050 scenarios corresponds to a 56.6% reduction in construction land within the high-susceptibility zone relative to the baseline (17.69 km^2^ vs. 7.67 km^2^, Table 5), far larger than the variability induced by parameter perturbation, which changes the simulated land-use area by less than 2% (Section 3.2.1 and Section 3.2.3). The scenario difference is therefore a deterministic consequence of the imposed constraint rather than an artifact of parameter settings or stochastic variation.

#### 3.3.2. Exposure Transfer Pathways: Evidence from Land-Use Transition Matrices

**Figure 6:**
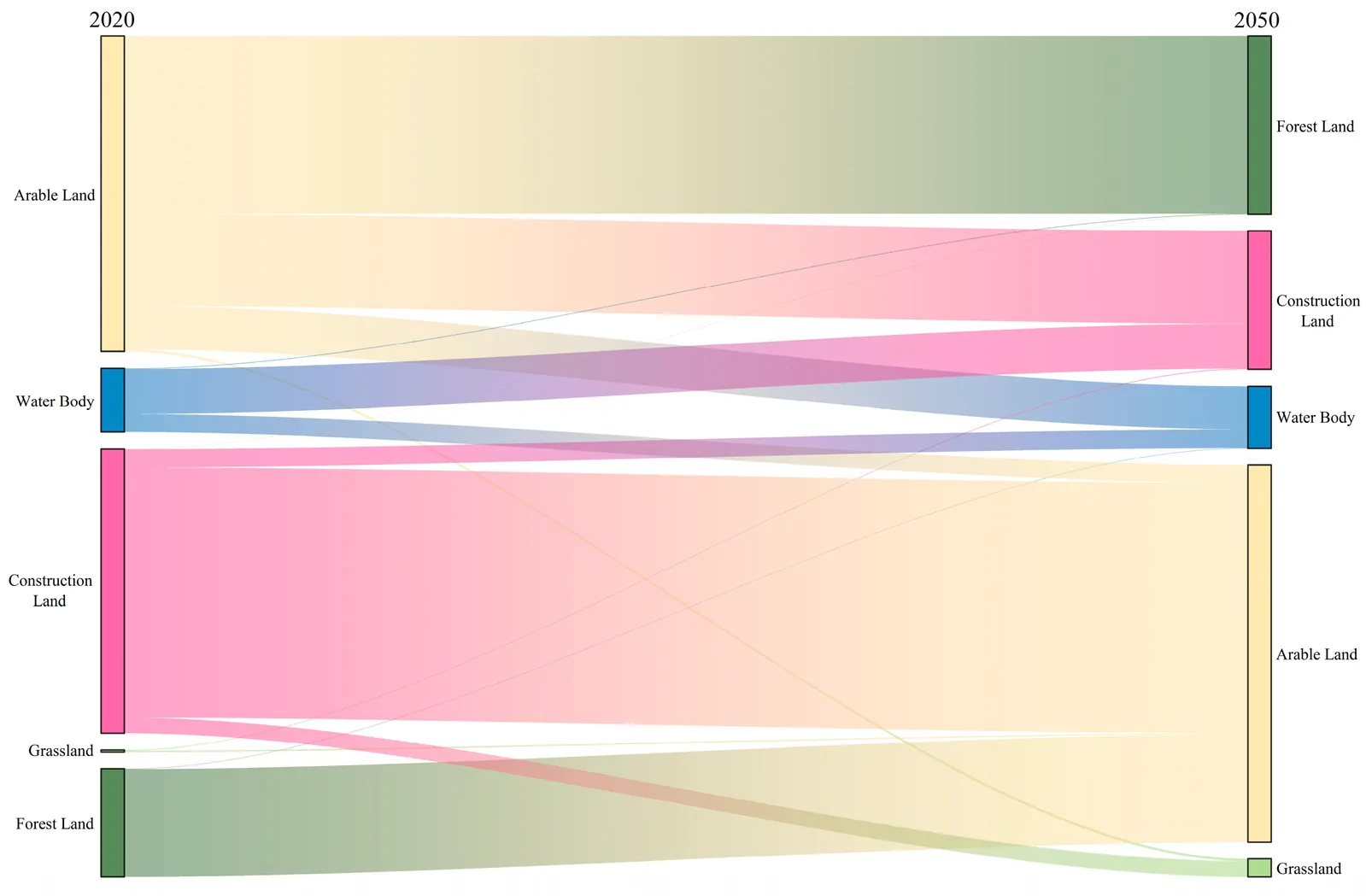
Sankey diagram of land-use transitions in the high-susceptibility zone under the susceptibility-constrained scenario (2020–2050).

Using land-use transfer matrices calculated separately for the high- and non-high-susceptibility zones under both the baseline and susceptibility-constrained scenarios, we identified distinct transition patterns. For the high-susceptibility zone, the baseline scenario projected substantial built-up encroachment, with arable land converting to construction land (9.28 km^2^) and to forest land (1.50 km^2^); by contrast, the susceptibility-constrained scenario sharply reduced this encroachment (arable land to construction land: 0.54 km^2^) and additionally converted 1.46 km^2^ of construction land back to arable land (built-up retreat), while arable land to forest land remained comparable (1.04 km^2^). Thus, the constrained scenario curbs the main landslide exposure pathway (arable land loss to construction within the high-susceptibility zone), and even retrofits part of the existing built-up area (Table 6). The complete set of net land-use flows within the high-susceptibility zone is visualized in Figure 6 (susceptibility-constrained scenario) and Figure S5 (baseline scenario). Development pressure displaced from the high-susceptibility zone is partly accommodated in the non-high-susceptibility zone: arable land to construction land there rises from 24.62 km^2^ (baseline) to 34.52 km^2^ (constrained), indicating that the constraint redirects rather than suppresses development. The full non-high-susceptibility zone transfer matrices are provided in Table S10.

#### 3.3.3. Ecosystem Service Value Assessment

The complete ESV accounting results, disaggregated by land-use types and susceptibility zones, are provided in Table S11. Table 7 shows that total ESV increased by approximately 11.5% from 2020 to 2050 under both scenarios (from CNY 424,497 × 10^4^ in 2020 to approximately CNY 473,000 × 10^4^), with nearly identical total values (CNY 473,336 vs. 472,922 × 10^4^, with difference < 0.1%). This indicates that susceptibility-constrained planning does not substantially reduce the estimated total ecosystem service value. However, the spatial distribution differs markedly. Under the susceptibility-constrained scenario, the proportion of total ESV located within high-susceptibility zones decreased from 8.03% to 7.25% (slightly lower than the baseline scenario’s 7.34%), representing a spatial shift of ecological value away from hazardous areas. At first glance, the baseline appears to achieve a larger ESV gain within high-susceptibility zones. However, as shown in Table 5, this gain is associated with a 107.47% increase in construction land within the same zone. This is a pattern where ecological gains are offset by concurrent construction expansion in the same hazardous zone, making the net ecological benefit questionable. In contrast, the susceptibility-constrained scenario’s smaller but intentional ecological gains occur under decreasing construction pressure, making them more durable. On the other hand, as can be deduced from Table 7, both scenarios achieved nearly identical ESV gains (CNY 438,602 vs. 438,628 × 10^4^) within the non-high-susceptibility zone, yet the susceptibility-constrained scenario supported substantially higher construction growth while maintaining the same ecological land gain (see Table 5), indicating greater land-use efficiency in geologically safe areas.

The ESV gain–loss map (Figure S6) reveals the spatial pattern of ecological benefits under the susceptibility-constrained scenario. Across the study area, 89.68 km^2^ shows positive ESV gains (green), indicating where this scenario preserves higher ecological value than the baseline scenario. Of these gains, 10.36 km^2^ (11.55%) are concentrated in the high-susceptibility zone, where the terrain is most vulnerable to landslides. Spatially, these 10.36 km^2^ are not randomly distributed. They predominantly occur along the Jialing River and its tributaries. These locations correspond to critical ecological contexts, particularly riverine buffers, where the simulated conversion of 1.04 km^2^ of arable land to forest (Table S10) and the retreat of construction land to arable land (1.46 km^2^) have enhanced both ESV and slope stability. The concentration of ESV gains in these specific locations is ecologically significant. Riverine forests and wetlands provide disproportionately high regulating services (including hydrological regulation, soil retention, and slope stabilization), which are precisely the functions that mitigate landslide susceptibility. By preserving 10.36 km^2^ of these high-value ecosystems within the high-susceptibility zone, the susceptibility-constrained scenario achieves a dual benefit: it maintains critical disaster-regulating services exactly where they are most needed, while avoiding the construction of new assets in hazardous areas.

In contrast, the red areas (where the baseline scenario preserves higher ESV) are scattered and predominantly located in non-high-susceptibility zone, representing marginal ecological gains that do not contribute to landslide susceptibility reduction. This spatial contrast further underscores the advantage of the susceptibility-constrained scenario: it strategically concentrates ecological benefits in high-susceptibility zones, whereas the baseline’s ecological advantages are located in areas where they provide limited hazard mitigation value.

## 4. Discussions

This study integrated landslide susceptibility into land-use simulation to test whether construction restrictions in high-susceptibility zones can reduce future landslide exposure without compromising ecosystem service value. The results support the central hypothesis: the susceptibility-constrained scenario reduces construction land within high-susceptibility zones by 56.6% relative to the baseline 2050 projection (7.67 vs. 17.69 km^2^; −10.01% relative to 2020), consistent with the hypothesized direction of the exposure reduction, while total ecosystem service value remains nearly unchanged (<0.1% difference). Below we interpret the observed susceptibility patterns, situate our approach within the broader literature, examine the policy implications and model uncertainties, and outline directions for future research.

### 4.1. Interpretation of Susceptibility Patterns

Although the northwest of Nanbu County primarily consists of hilly terrain, it houses the largest local reservoir, Shengzhong Reservoir, and Shengzhong Lake National Wetland Park. The construction of the wetland ecological park effectively protects the region’s ecological security, resulting in extensive forest coverage and consequently lower landslide susceptibility. The central region, while relatively flat, is heavily influenced by human activities. Along the river, construction land and large tracts of farmland are more susceptible to landslides during the rainy season. Therefore, future development should emphasize disaster prevention, assessing landslide susceptibility when planning layouts, avoiding very high-susceptibility-level areas, and considering and enhancing disaster prevention safety during construction. These spatial patterns are quantitatively consistent with the class-resolved factor analysis reported in Table 2, where the very low-susceptibility class is dominated by high elevation and lake areas, whereas the very high class is characterized by urban lithology, newly deposited and yellow soils, and high annual precipitation.

### 4.2. Comparison with Previous Studies

In the last decade, a significant increase in landslides and other geological disasters in China has led to alterations in local land-use patterns. The research addressing these events typically revolves around two primary aspects. First, the examination of the relationship between geological disasters and land use. Xie et al. used remote sensing interpretation and GIS analysis to investigate the sensitivity of various land-use types in the Xiajiang Basin to landslides and determined their impact severity [55]. Secondly, there is a growing body of research on evaluating the impact of these events and developing subsequent land-use planning strategies. Jia et al. conducted research on landslide-affected lands in the Huangtou District of Tianshui City, explored the temporal–spatial patterns of land-use changes from 1985 to 2020, providing guidance for the planning and development of affected areas [21]. In summary, scholars from both national and international domains have delved into understanding landslides and related geologic hazards, land use, and their linkages, but there remains a need for more in-depth analysis on the impacts on land use and development of methods and planning measures for land management adjustments, which we aim to address in our study.

In land-use simulation research, scholars often explore land-use changes from the perspectives of economic development, ecological protection, and arable land protection, with some considering the impact of transportation, infrastructure development, and planning strategies [56]. Xie et al. conducted multi-scenario land-use simulations for Nanchuan District, Chongqing, based on the PLUS model [57]. The results showed that construction land had the highest spatial aggregation effect along the central urban area in the economic development scenario, which aligns with the baseline scenario results in this study, indicating that scenarios without constraints tend towards economic development. Other multi-scenario simulation studies have also shown that ecological red lines and permanent basic farmland limit the unregulated expansion of construction land, helping to ensure regional ecological functions and food security. Around the world, there are numerous cities that are severely affected by landslide susceptibility, similar to Nanbu County in Nanchong City, Sichuan Province, China. For example, in some mountainous regions with abundant rainfall, such as Mumbai in India and Medellín in Colombia, their topographical and climatic conditions contribute to a persistently high landslide susceptibility. In Hong Kong, China, over 100,000 mountain landslides have occurred in the past few decades, resulting in losses amounting to hundreds of millions of Hong Kong dollars annually [58]. In Gong County, Yibin City, Sichuan Province, China, based on field investigations of geological hazards, a total of 136 landslide disasters have been identified within the territory of Gong County, accounting for 56.44% of the total number of all geological disasters in the county. This has posed serious safety threats to the lives and property of local residents. When conducting land-use planning, these cities are in urgent need of incorporating the landslide susceptibility into consideration. By means of precise geological exploration, scientific susceptibility assessment, and the rational layout of land use, it is possible to reduce the potential losses that may be brought about by landslide disasters. Avoiding landslide susceptibility during land planning and development in geologically susceptible areas is equally important. Related research on land-use simulations incorporating landslide susceptibility is relatively sparse.

### 4.3. Policy Implications

The susceptibility-constrained scenario corresponds to China’s ‘Three Zones and Three Lines’ territorial spatial planning framework. The high-susceptibility zone, where construction is restricted, parallels the Ecological Protection Red Line, which designates areas for strict ecological conservation. The non-high-susceptibility zone, where development is permitted, corresponds to the Urban Development Boundary and Permanent Basic Farmland Protection Line. Incorporating landslide susceptibility as an additional constraint layer adds a susceptibility-informed dimension missing from the current ‘Three Zones and Three Lines’ designation process. This integration could strengthen the scientific basis of territorial spatial planning in landslide-prone mountainous regions.

### 4.4. Uncertainty, Limitations, and Future Research

We noted that coupling the CF-LR, Markov, and PLUS models propagates uncertainty into the final simulations: CF-LR susceptibility layer and threshold errors most directly affect where development is permitted within the high-susceptibility zone, whereas Markov demand errors shift each land-use total. Independent 2020 validation of each module and a PLUS parameter sensitivity analysis help to bound this propagated uncertainty for the retained parameter settings, although the sensitivity of the FOM to the neighborhood range (Section 3.2.1) means that cell-scale agreement is conditional on the calibrated NS value; neither source of variability overturns the principal finding that the constrained scenario curbs high-susceptibility zone encroachment.

This study has several limitations. First, the landslide inventory is limited. There is less information about historical landslide disaster points in the northwestern hilly areas of Nanbu County, possibly because the regional topography makes it difficult to obtain data. The AUC of 0.708 indicates moderate predictive power, likely reflecting the limited number of historical landslide points and the inherent complexity of landslide-driving factors. Reichenbach et al. noted that AUC values in statistically based landslide susceptibility models vary widely depending on inventory size, data quality, and terrain complexity [6]. While the model captures the general spatial pattern of susceptibility, it may not fully resolve local-scale variations.

Second, a single temporal validation point (2020) is insufficient to fully characterize the model’s predictive reliability over multi-decadal horizons. Future research should incorporate additional validation years as new land-use data become available.

Third, the conclusions reflect the performance of a specific susceptibility-constrained scenario compared to a baseline scenario. We did not evaluate alternative policy options such as partial restrictions, engineering measures, priority ecological restoration, or compact urban development. A comprehensive evaluation of multiple management strategies would be necessary to identify the optimal land-use planning approach.

Fourth, the ESV assessment employs the equivalent factor method, which standardizes but simplifies the monetization of ecosystem services. This approach does not capture spatial heterogeneity within land-use classes, does not account for service flows or demand, and relies on static coefficients that may not reflect future socioeconomic conditions. The method also omits biodiversity, ecological connectivity, and hydrological stability, components that are critical but difficult to monetize. The comparable total ESV between scenarios does not indicate equivalent overall sustainability.

Fifth, the CF-LR regression coefficients and susceptibility thresholds derived in this study are specific to the geological, climatic, and land-use conditions of Nanbu County. While the methodological framework is transferable, the model parameters should be recalibrated using local historical landslide data. Meanwhile, residual spatial autocorrelation in the CF-LR model may modestly inflate the reported AUC, as suggested by spatially blocked cross-validation. Nonetheless, the blocked CV AUC remains well above chance, indicating that the susceptibility classification retains clear value for land-use planning.

Several priorities for future research emerge from this study. (1) Incorporating climate change scenarios to account for shifts in precipitation and temperature; (2) quantifying and propagating model uncertainty through the coupled modeling chain; (3) integrating hydrological processes that influence slope stability; (4) conducting independent temporal validations as new land-use data become available; (5) evaluating multiple land-use policy scenarios, including moderate restrictions, engineering measures, and compact development; and (6) extending the framework to incorporate exposure and vulnerability analysis for full disaster risk assessment.

## 5. Conclusions

By coupling the CF-LR model with the PLUS model, this research provides a new framework that can be adapted to different regions, considering the impact of landslide susceptibility on the spatial distribution of land use. The key findings drawn from this case study of Nanbu County, Sichuan Province are summarized as follows:

(1) The CF-LR model proves effective for assessing landslide susceptibility, demonstrating sufficient accuracy. The PLUS model successfully simulates land-use changes, and their integration enables comprehensive land-use planning from a landslide susceptibility perspective.
(2) Landslide susceptibility varies across different regions, with high-susceptibility zones more concentrated in central locations, while non-high-susceptibility zones are typically found in the western and northwestern regions. This distribution can inform land-use strategies to mitigate landslide susceptibility.
(3) The land-use trend from 2020 to 2050 primarily shows a shift from arable land to forest and construction land. Urban and town construction land is expected to expand outward, while forest land will grow significantly in some areas, reflecting broader ecological and development policies.
(4) Under a baseline scenario, construction land expansion near urban areas shows a clustering pattern. However, when considering landslide susceptibility, the expansion avoids high-susceptibility zones, indicating the need for future development plans to prioritize disaster prevention and avoid restricted areas prone to geohazards.
(5) The susceptibility-constrained scenario delivers demonstrable ecological co-benefits without sacrificing overall ecosystem service value. Relative to the baseline 2050 simulation, construction land within high-susceptibility zones is 56.6% lower under the susceptibility-constrained scenario (7.67 vs. 17.69 km^2^), and it even declines by 10.01% relative to 2020 (versus a 107.47% increase under the baseline), consistent with the hypothesized direction of the reduction in landslide exposure. The ESV gain–loss mapping shows that 10.36 km^2^ of the preserved high-value areas are located within high-susceptibility zones, primarily comprising riverine forests and wetlands. Transfer matrix analysis further indicates that the ecological gains within hazardous zones arise from the modeled conversion of arable land to forest (1.04 km^2^) and the retreat of construction land to arable land (1.46 km^2^) under the susceptibility-constrained scenario, in contrast with the abandonment processes dominating the baseline scenario.

The principal scientific advancement of this study is the explicit coupling of landslide susceptibility assessment with land-use simulation under a controlled experimental design, enabling an early quantitative evaluation of whether construction restrictions in high-susceptibility zones can reduce future landslide exposure while maintaining ecosystem service value. Unlike previous studies that assessed susceptibility or simulated land-use change largely in isolation, this framework integrates landslide susceptibility as a spatial constraint, providing a transferable methodology for susceptibility-informed land-use planning in mountainous regions.

In summary, this study demonstrates that integrating landslide susceptibility into land-use planning does not necessitate a trade-off between disaster exposure reduction and ecological conservation. The susceptibility-constrained scenario achieves comparable overall ecosystem service value while substantially reducing disaster exposure and concentrating the modeled conversion toward forest within hazardous zones. By coupling landslide susceptibility assessment, land-use simulation, and ESV evaluation, this framework provides a practical, transferable approach for regions prone to geological hazards, supporting safer urban development while maintaining the estimated ecosystem service value, within the scope of the analyses presented here.

## Figures and Tables

**Table 2:** Dominant environmental factors associated with the five landslide susceptibility classes.

Susceptibility Class	Dominant Factor(s)	Representative Class (CF)	Secondary Factor(s)
Very high	Surface lithology; soil type	Urban area (0.676); basalt (0.589); newly deposited soil (0.546); yellow soil (0.537)	Precipitation 1049–1066 mm (0.453); NDVI 0.54–0.69 (0.453)
High	Annual precipitation; surface lithology	1049–1066 mm (0.453); 1066–1088 mm (0.364); Shale (0.472)	Slope 20–30° (0.125); west/SW aspect (0.185/0.181)
Moderate	Slope; aspect	Slope 5–10° (0.106); 10–20° (0.055); west aspect (0.185)	Distance to water < 900 m (0.113)
Low	Elevation; slope	Elevation 485–566 m (−0.228); slope 0–5° (−0.310); NDVI 0.76–0.82 (−0.172)	Distance to road 1500–2500 m (−0.290)
Very low	Elevation; soil/distance to water	Elevation 566–818 m (−0.453); lake (−0.717); distance to water > 4900 m (−0.749)	Temperature 15.7–16.2 °C (−0.520)

Note: CF denotes the certainty factor; positive values indicate a positive association with landslide occurrence and negative values a negative association. The dominant factor(s) for each susceptibility class are identified from the factor classes with the largest CF magnitudes in Table S4; the complete CF values for all factor classes are listed therein.

**Table 3:** The contribution of different driving factors to the change of each land-use type.

Category	Arable Land	Forest Land	Grassland	Water Body	Construction Land
Elevation	0.134	0.108	0.001	0.410	0.067
Slope	0.078	0.064	0.007	0.090	0.043
Aspect	0.036	0.044	0.004	0.061	0.020
Soil type	0.003	0.002	0.000	0.075	0.010
Annual precipitation	0.219	0.257	0.202	0.008	0.017
Annual average temperature	0.091	0.045	0.059	0.030	0.050
Population	0.093	0.098	0.069	0.102	0.264
GDP	0.077	0.097	0.544	0.028	0.129
Distance to primary road	0.100	0.084	0.029	0.036	0.023
Distance to secondary road	0.048	0.041	0.058	0.032	0.027
Distance to tertiary road	0.063	0.088	0.019	0.067	0.322
Distance to water system	0.057	0.071	0.009	0.062	0.028

**Table 4:** Predicted land-use demand by land-use types (2025–2050).

Year	Land-Use Demand in Number of Grid Cells				
	Arable Land	Forest Land	Grassland	Water Body	Construction Land
2025	1,991,201	352,506	99	75,586	40,167
2030	1,950,025	387,062	64	76,094	46,314
2035	1,915,015	415,632	49	76,679	52,185
2040	1,885,149	439,214	42	77,337	57,816
2045	1,859,578	458,642	39	78,065	63,235
2050	1,837,590	474,608	38	78,862	68,461

Note: the 2025–2045 values are independently analyzable intermediate Markov demand estimates, and the spatial comparison is deliberately centered on the 2050 terminal state.

**Table 5:** Land-use dynamics within and outside high-susceptibility zones (2020–2050).

Land-Use Type	Observed in 2020 (km^2^)	Baseline Scenario		Susceptibility-Constrained Scenario	
		2050 (km^2^)	Change (%)	2050 (km^2^)	Change (%)
**High-susceptibility zone**					
Arable land	86.30	75.80	−12.17	86.66	0.41
Forest land	1.99	2.93	47.55	2.40	20.61
Grassland	0.07	0.02	−76.41	0.16	130.61
Water body	10.15	10.60	4.47	10.14	−0.08
Construction land	8.52	17.69	107.47	7.67	−10.01
**Non-high-susceptibility zone**					
Arable land	1756.81	1578.93	−10.13	1568.02	−10.75
Forest land	272.03	424.37	56.00	424.81	56.16
Grassland	0.07	0.007	−91.11	0.005	−93.83
Water body	57.22	60.22	5.23	60.51	5.75
Construction land	21.22	43.85	106.63	54.01	154.53

Note: change rate was calculated from the unrounded area data; minor differences may arise if recalculated from the rounded values presented in the tables.

**Table 6:** Key land-use transitions in the high-susceptibility zone under baseline and susceptibility-constrained scenario (2020–2050).

Scenario	Transition	Area (km^2^)
Baseline	Arable land → forest land	1.50
	Arable land → water body	0.70
	Arable land → construction land	9.28
	Forest land → arable land	0.56
Susceptibility-constrained	Arable land → construction land	0.54
	Forest land → arable land	0.63
	Construction land → arable land	1.46
	Arable land → water body	0.70

Note: only transitions > 0.5 km^2^ are shown.

**Table 7:** Ecosystem service value comparison between scenarios (CNY ×10^4^).

Year and Scenario	Total ESV	ESV Within High-Susceptibility Zone	% of Total ESV
2020	424,497	34,100	8.03
2050 baseline	473,336	34,734	7.34
2050 susceptibility-constrained	472,922	34,294	7.25

## Data Availability

The original contributions presented in this study are included in the article/Supplementary Material. Further inquiries can be directed to the corresponding authors.

## Supplementary Materials

The following supporting information can be downloaded at: https://www.mdpi.com/article/10.3390/biology15181588/s1, Figure S1. Empirical semivariogram of the CF-LR residuals with the fitted spherical model. Figure S2. Landslide probability distribution map. Figure S3. Susceptibility-constrained simulated 2020 maps under the 20 single-parameter perturbations. Figure S4. Susceptibility-constrained simulated 2050 maps under the seven parameter-perturbation schemes (N1–N7) and the susceptibility-constrained 2050 simulation reported in the main text (Base). Figure S5. Sankey diagram of land use transitions in the high-susceptibility zone under the baseline scenario (2020–2050). Figure S6. ESV gain–loss map of Nanbu County showing difference in ESV between the two 2050 scenarios (susceptibility-constrained minus baseline). Table S1. Sown Area, Production, and Average Price in Nanbu County, 2020. Table S2. Ecosystem service equivalent values per unit area for each land use type in Nanbu County. Table S3. Ecosystem service value per unit area for each land use type in Nanbu County(CNY/hm^2^). Table S4. The deterministic coefficients of each evaluation factors. Table S5. Logistic regression results for landslide susceptibility factors. Table S6. Global Moran’s I of the CF-LR residuals under alternative spatial-weight specifications. Table S7. Results of spatially blocked cross-validation for the CF-LR model. Table S8. One-factor-at-a-time sensitivity of the PLUS model in the model calibration stage. Table S9. Long-term stability of the 2050 susceptibility-constrained simulation. Table S10. Land use transfer matrix under baseline scenario and susceptibility-constrained scenario (hm^2^). Table S11. Ecosystem service value disaggregated by land use type and susceptibility zone in Nanbu County (10^4^ CNY).

## Abbreviations

The following abbreviations are used in this manuscript:

PLUS	Patch-generating Land Use Simulation
CF-LR	Certainty factor–logistic regression
ESV	Ecosystem Service Value
SD	System Dynamics
CA	Cellular Automata
FLUS	Future Land Use Simulation
GIS	Geographic Information System
AHP	Analytic Hierarchy Process
SVM	Support Vector Machine
RF	Random Forest
DEM	Digital Elevation Model
GDP	Gross Domestic Product
NDVI	Normalized Difference Vegetation Index
CARS	CA based on Multiple Random Seeds
FOM	Figure of Merit
LEAS	Land Expansion Analysis Strategy
ROC	Receiver Operating Characteristic
AUC	Area Under the Curve
TOL	Tolerance
VIF	Variance Inflation Factor

## References

[1] Petley D. (2012). Global patterns of loss of life from landslides. Geology.

[2] Ozturk U., Bozzolan E., Holcombe E.A., Shukla R., Pianosi F., Wagener T. (2022). How climate change and unplanned urban sprawl bring more landslides. Nature.

[3] Ministry of Natural Resources of China (2021). Bulletin of National Geological Hazards (2011–2020).

[4] Li B.V., Jenkins C.N., Xu W. (2022). Strategic protection of landslide vulnerable mountains for biodiversity conservation under land-cover and climate change impacts. Proc. Natl. Acad. Sci. USA.

[5] Glade T. (2003). Landslide occurrence as a response to land use change: A review of applied approaches. Catena.

[6] Reichenbach P., Rossi M., Malamud B.D., Mihir M., Guzzetti F. (2018). A review of statistically-based landslide susceptibility models. Earth Sci. Rev..

[7] Kuang W.H., Zhang S.W., Du G.M., Yan C.Z., Wu S.X., Li R.D., Lu D.S., Pan T., Ning J., Guo C.Q. (2022). Remotely sensed mapping and analysis of spatio—Temporal patterns of land use change across China in 2015–2020. Acta Geogr. Sin..

[8] He S.L., He Z., Pan J., Wang J. (2023). County land use/land cover simulation based on Multi-Model. Remote Sens. Nat. Resour..

[9] Zeng T., Guo Z., Wang L., Jin B., Wu F., Guo R. (2023). Tempo-spatial landslide susceptibility assessment from the perspective of human engineering activity. Remote Sens..

[10] Promper C., Puissant A., Malet J.-P., Glade T. (2014). Analysis of land cover changes in the past and the future as contribution to landslide risk scenarios. Appl. Geogr..

[11] Luan C., Liu R., Li Y., Zhang Q. (2024). Comparison of various models for multi-scenario simulation of land use/land cover to predict ecosystem service value: A case study of Harbin-Changchun Urban Agglomeration, China. J. Clean. Prod..

[12] Li Q., Wang C., Feng T., Du C., Zhang B. (2024). Multi-Scenario Prediction of Land Use Change and Carbon Storage in Shaanxi Province Based on the SD-PLUS Coupled Model. J. Soil Water Conserv..

[13] Wei J.B., Zhao Z. (2018). Analysis of geological hazard susceptibility based on the weighted certainty factor method. Coal Geol. Explor..

[14] Yuan X., Liu C., Nie R., Yang Z., Li W., Dai X., Cheng J., Zhang J., Ma L., Fu X. (2022). A Comparative Analysis of Certainty Factor-Based Machine Learning Methods for Collapse and Landslide Susceptibility Mapping in Wenchuan County, China. Remote Sens..

[15] Zhang Z., Sun J. (2024). Regional Landslide Susceptibility Assessment and Model Adaptability Research. Remote Sens..

[16] Xie G.D., Zhang C., Zhang L., Chen W., Li S. (2015). Improvement of the Evaluation Method for Ecosystem Service Value Based on Per Unit Area. J. Nat. Resour..

[17] Costanza R., de Groot R., Sutton P., van der Ploeg S., Anderson S.J., Kubiszewski I., Farber S., Turner R.K. (2014). Changes in the global value of ecosystem services. Glob. Environ. Chang..

[18] Xie G., Zhang C., Zhen L., Zhang L. (2017). Dynamic changes in the value of China’s ecosystem services. Ecosyst. Serv..

[19] Luan C., Liu R. (2022). A Comparative Study of Various Land Use and Land Cover Change Models to Predict Ecosystem Service Value. Int. J. Environ. Res. Public Health.

[20] Sidle R.C., Ochiai H. (2006). Landslides: Processes, Prediction, and Land Use.

[21] Jia J., Su X., Zhang J., Zhang M., Li X., Wei W. (2023). Spatial and Temporal Variation Characteristics of Landslide Disaster Damage Land Use in Loess Area of Tianshui City from 1985 to 2020. J. Soil Water Conserv..

[22] Jurchescu M., Kucsicsa G., Micu M., Bălteanu D., Sima M., Popovici E.-A. (2023). Implications of future land-use/cover pattern change on landslide susceptibility at a national level: A scenario-based analysis in Romania. Catena.

[23] Zhao F., Miao F., Wu Y., Gong S., Zheng G., Yang J., Zhan W. (2024). Landslide dynamic susceptibility mapping in urban expansion area considering spatiotemporal land use and land cover change. Sci. Total Environ..

[24] Yang C., Wang J., Li S. (2024). Landslide Susceptibility Assessment and Future Prediction with Land Use Change and Urbanization Towards Sustainable Development: The Case of the Li River Valley in Yongding, China. Sustainability.

[25] Sichuan Provincial Bureau of Statistics Sichuan Statistical Yearbook 2023. https://tjj.sc.gov.cn/scstjj/tjnjnew/2023/zk/indexeh.htm.

[26] Zhang H.X., Sun Q., Fan S., Yang Z. (2023). Evaluation Methods and Accuracy Comparison of Landslide Susceptibility—Dafang County of Guizhou Province Is Taken as an Example. Guizhou Geol..

[27] Geographic Remote Sensing Ecological Network. National Geological Hazard Points Spatial Distribution Data. https://www.gisrs.cn/?data_172/52c30b65-7214-422e-9e89-eeb101b49561.html.

[28] Yang J., Huang X. (2022). The 30 m Annual Land Cover Datasets and Its Dynamics in China from 1990 to 2021.

[29] ASTER GDEM V2 30 m Digital Elevation Data. Geospatial Data Cloud, Computer Network Information Center, Chinese Academy of Sciences.

[30] China Soil Type Spatial Distribution Data (1:1,000,000). Resource and Environmental Science Data Center, Chinese Academy of Sciences. https://www.resdc.cn/data.aspx?DATAID=145.

[31] Xu X. (2017). China GDP Spatial Distribution Kilometer Grid Dataset.

[32] Bondarenko M., Priyatikanto R., Tejedor-Garavito N., Zhang W., McKeen T., Cunningham A., Woods T., Hilton J., Cihan D., Nosatiuk B. (2025). Constrained Estimates of 2015–2030 Total Number of People Per Grid Square at a Resolution of 3 Arc (Approximately 100 m at the Equator), R2025A Version v1.

[33] Fick S.E., Hijmans R.J. (2017). WorldClim 2: New 1-km spatial resolution climate surfaces for global land areas. Int. J. Clim..

[34] China Annual Vegetation Index (NDVI) Spatial Distribution Dataset. Geographic Remote Sensing Ecological Network. https://www.gisrs.cn/?data_157/05b59e69-ba30-4454-a9c0-67ca038fb9f3.html.

[35] Dijkshoorn J.A., van Engelen V.W.P., Huting J.R.M. Soil and Terrain Database for China (SOTER_China, Version 1.0).

[36] OpenStreetMap Database. OpenStreetMap Contributors. https://www.openstreetmap.org.

[37] Liu Y., Wang N., Zhou C., Xie J., Li Y. (2020). Evaluation of Landslide Susceptibility Based on ROC and Certainty Factor Method in Fengjie County, Three Gorges Reservoir. Saf. Environ. Eng..

[38] Qin H.F., Tan S., Shi Y., Li H., Wang B. (2022). Geological hazard susceptibility assessment based on CF&LR combined model: Case of Ning’er Hani and Yi Autonomous County, Yunnan Province. Yangtze River.

[39] Luo L.G., Pei X., Huang R., Pei Z., Zhu L. (2021). Landslide susceptibility assessment in Jiuzhaigou scenic area with GIS based on certainty factor and Logistic regression model. J. Eng. Geol..

[40] Wang X., Shi Y., Chen H. (2022). Evaluation of Geological Hazard Susceptibility Based on certainty factors Coupling logistic regression. Bull. Surv. Mapp..

[41] Tu S.Y., Zhang Z., Fu H., Xu S., Deng M., He L., Liu J. (2022). Geological hazard susceptibility evaluation based on CF and CF-LR model. Chin. J. Geol. Hazard Control.

[42] Qin Y.G., Yang G., Jiang X., Lu K., Li Z. (2020). Geohazard susceptibility assessment based on integrated certainty factor model and logistic regression model for Kaiyang, China. Sci. Technol. Eng..

[43] Li X.Q., Liang Y.L. (2022). Land Use Response Mechanism Caused by Geological Disasters: Taking Mianzhu City As an example. Northwest. Geol..

[44] Lucchese L.V., de Oliveira G.G., Pedrollo O.C. (2021). Investigation of the influence of nonoccurrence sampling on landslide susceptibility assessment using Artificial Neural Networks. Catena.

[45] Zhang J.Y., Ding Y., Sun D. (2022). Landslide susceptibility evaluation based on different sample proportion and super parameter optimization: Take Wulong district of Chongqing municipality as an example. J. Chongqing Norm. Univ. Nat. Sci..

[46] Liu Y.H., Fang R., Su Y., Xiao R. (2021). Machine learning based model for warning of regional landslide disasters. J. Eng. Geol..

[47] Yang S., Su H., Zhao G. (2022). Multi-Scenario Simulation of Urban Ecosystem Service Value Based on PLUS Model: A Case Study of Hanzhong City. J. Arid Land Resour. Environ..

[48] Zhi F., Zhou Z., Zhao M., Wang S. (2024). Temporal and Spatial Evolution Characteristics of Carbon Storage in Hefei Ecosystem Based on PLUS and InVEST Models. J. Soil Water Conserv..

[49] Wang N.Q., Guo Y., Liu T., Zhu Q. (2019). Assessment of landslide susceptibility based on SVM-LR model: A case study of Lintong district. Sci. Technol. Eng..

[50] Wang J.N., Wang W., Hai M. (2022). Simulation Analysis of Land Use Change in Shandong Province Based on PLUS Model. Territ. Nat. Resour. Study.

[51] (2021). Nanchong Statistical Yearbook 2021. Nanchong Municipal Statistical Yearbook. Compiled by Nanchong Municipal Bureau of Statistics. https://www.nanchong.gov.cn/zwgk/sjfb/tjnj/t_889034.html.

[52] (2023). The Third National Land Survey Main Data Bulletin of Nanbu County. Nanbu County People’s Government. https://www.scnanbu.gov.cn/xwdt/ztzl/jczwgk/gkly/zrzyly/dcjc/dlgqjccg/202312/t20231206_1908645.html.

[53] (2021). Southern County Forest Fire Prevention and Extinguishing Has “Thousand-Mile Eye”. Nanchong Municipal Development and Reform Commission. https://www.nanchong.gov.cn/fgw/xwdt/qxdt/202104/t20210414_1590646.html.

[54] Han L.Y., Cai Q.G. (2004). A Brief Discussion on the Impact of Land Use on Landslides. Soil Water Conserv. Sci. Technol. Shanxi.

[55] Xie X.J., Wei F., Lan D. (2011). Sensitivity of landslide to land use in Xiaojiang River Basin. J. Nat. Disasters.

[56] Zhao X., Peng J., Fan Z., Yang C., Yang H. (2020). Land use simulation and urban growth boundaries delineation in Wuhan metropolitan area based on FLUS model and “Dual Environment Evaluation”. J. Geo Inf. Sci..

[57] Xie X.D., Lin X., Wang Y., Tu R., Zhang J. (2023). Multi-scenario Simulation of Land Use in Nanchuan District of Chongqing Based on PLUS Model. J. Yangtze River Sci. Res. Inst..

[58] Li W.M., Lo F.L.C., Wong T.K.C., Cheung R.W.M. (2022). Machine Learning-Powered Rainfall-Based Landslide Predictions in Hong Kong-An Exploratory Study. Appl. Sci..

