# Supplementary material for: Effect of Construction Restrictions on Future Landslide Exposure Under Land-Use Simulation in Southwest China
Source: Biology (Basel). 2026 Sep 9;15(18):1588. doi: 10.3390/biology15181588 (PMC13604593; doi:10.3390/biology15181588)
Supplement: Supplementary file 1 [file biology-15-01588-s001.zip › biology-4438881-supplementary.pdf]

Supplementary Materials (SI)

Effect of Construction Restrictions on Future Landslide Expo-sure under Land-Use Simulation in Southwest China

A. Supplementary figures

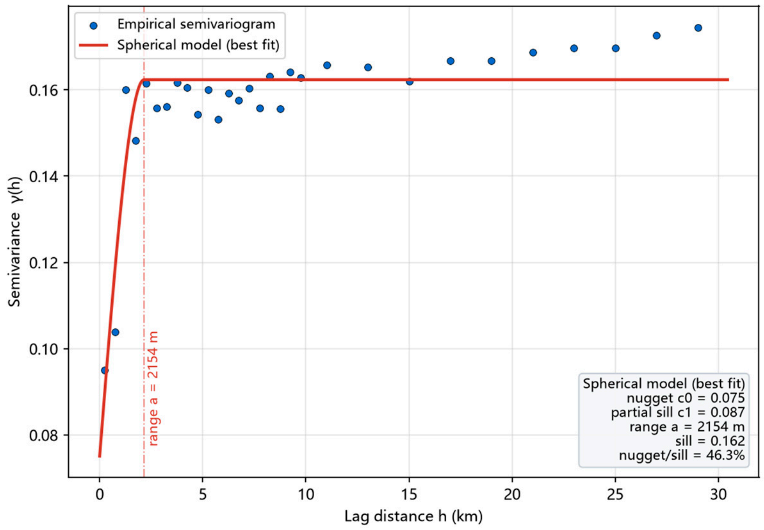

Figure S1. Empirical semivariogram of the CF-LR residuals with the fitted spherical model.

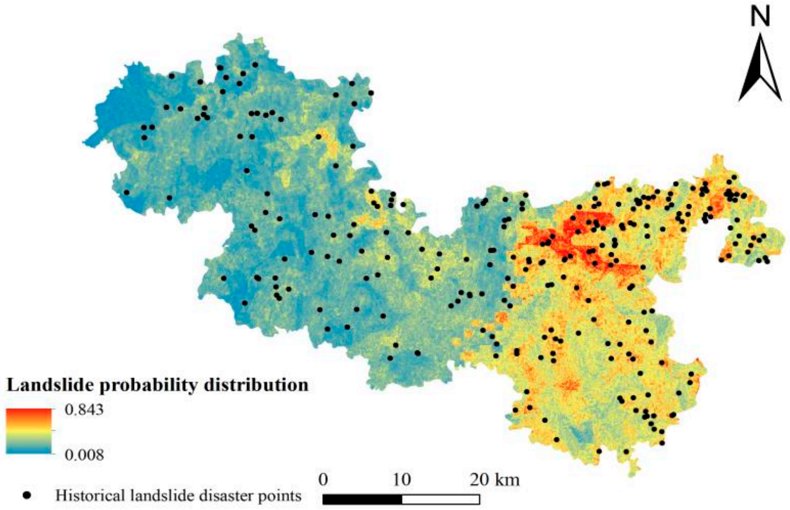

Figure S2. Landslide probability distribution map

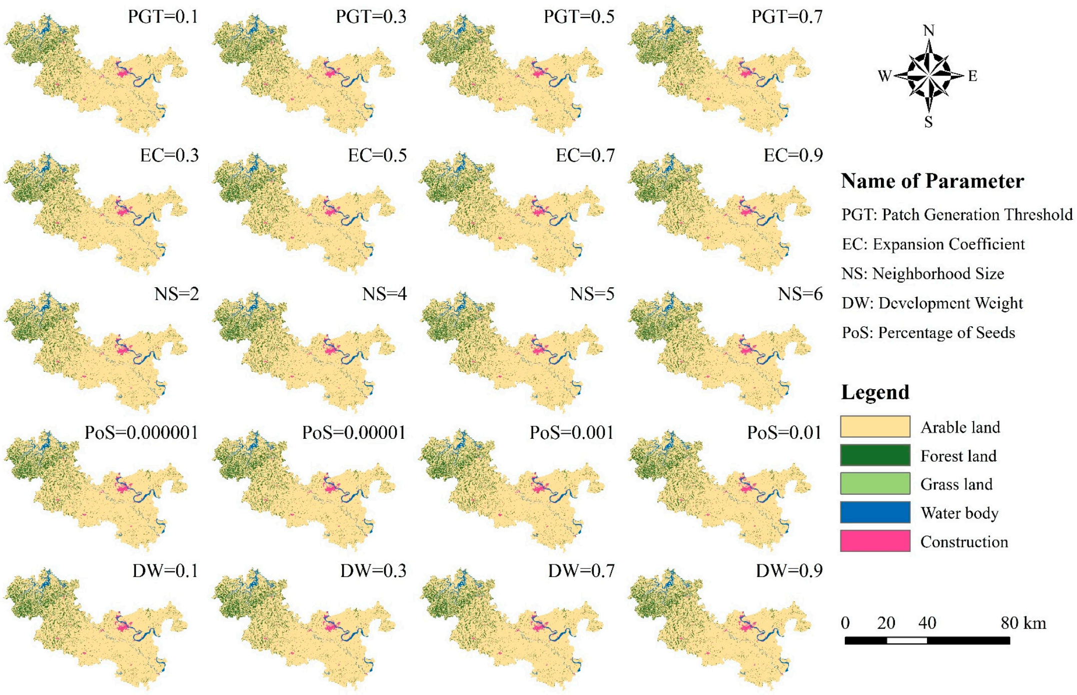

Figure S3. Susceptibility-constrained simulated 2020 maps under the 20 single-parameter perturbations.

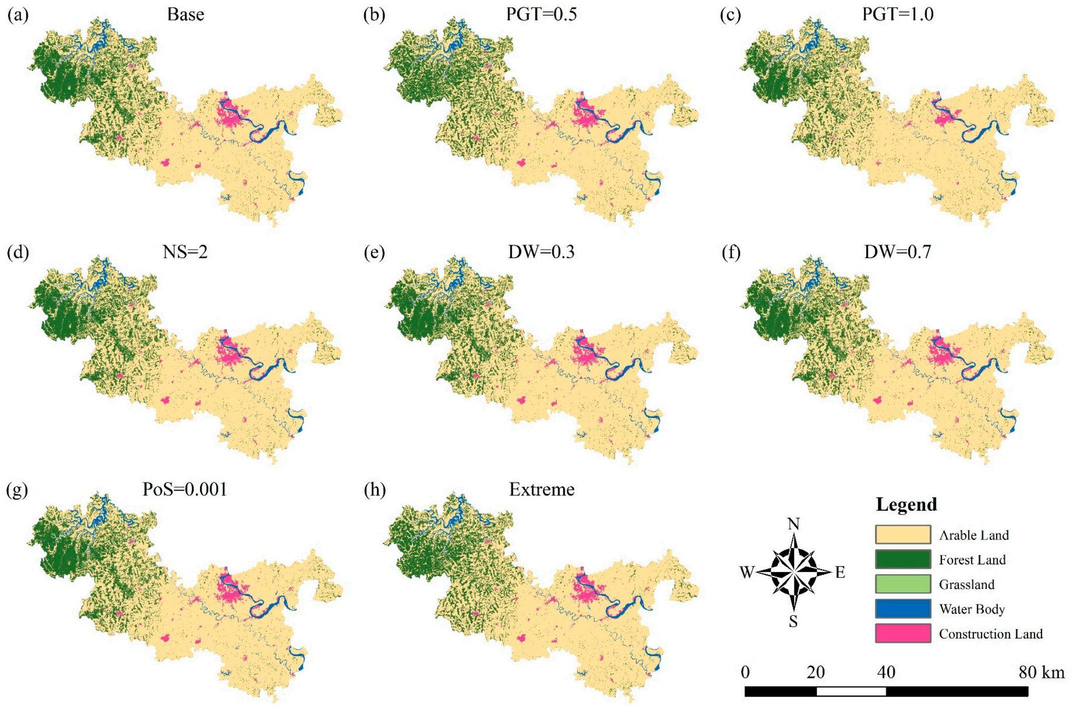

Figure S4. Susceptibility-constrained simulated 2050 maps under the seven parameter-perturbation schemes (N1–N7) and the susceptibility-constrained 2050 simulation reported in the main text (Base).

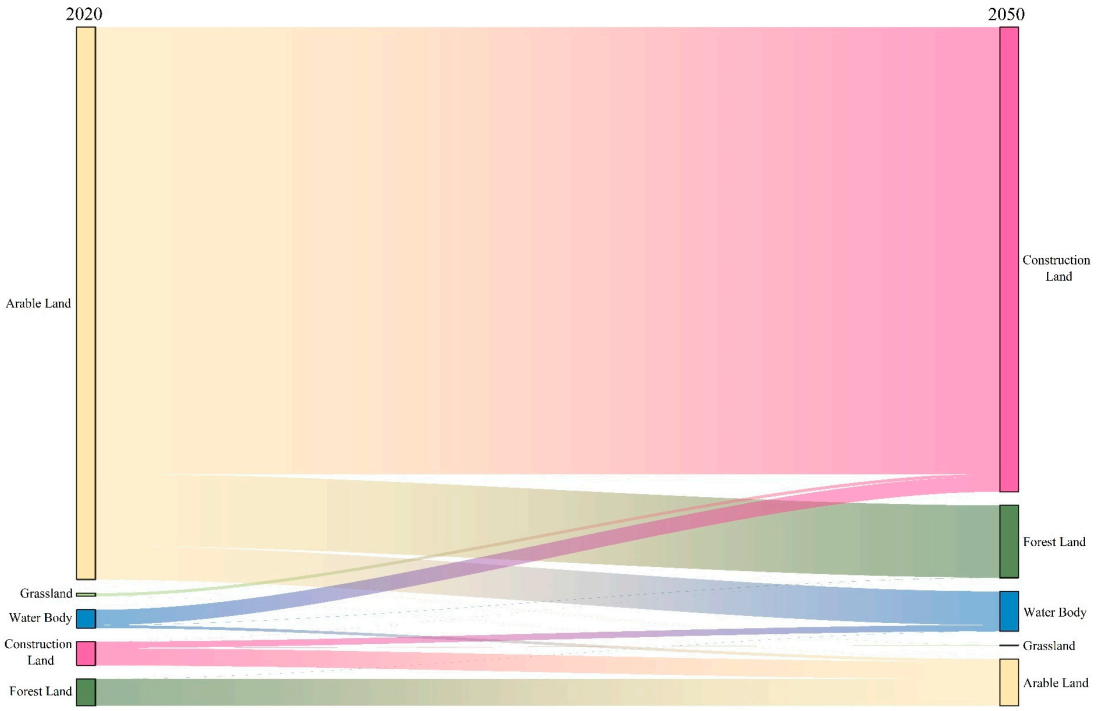

Figure S5. Sankey diagram of land use transitions in the high-susceptibility zone under the baseline scenario (2020–2050).

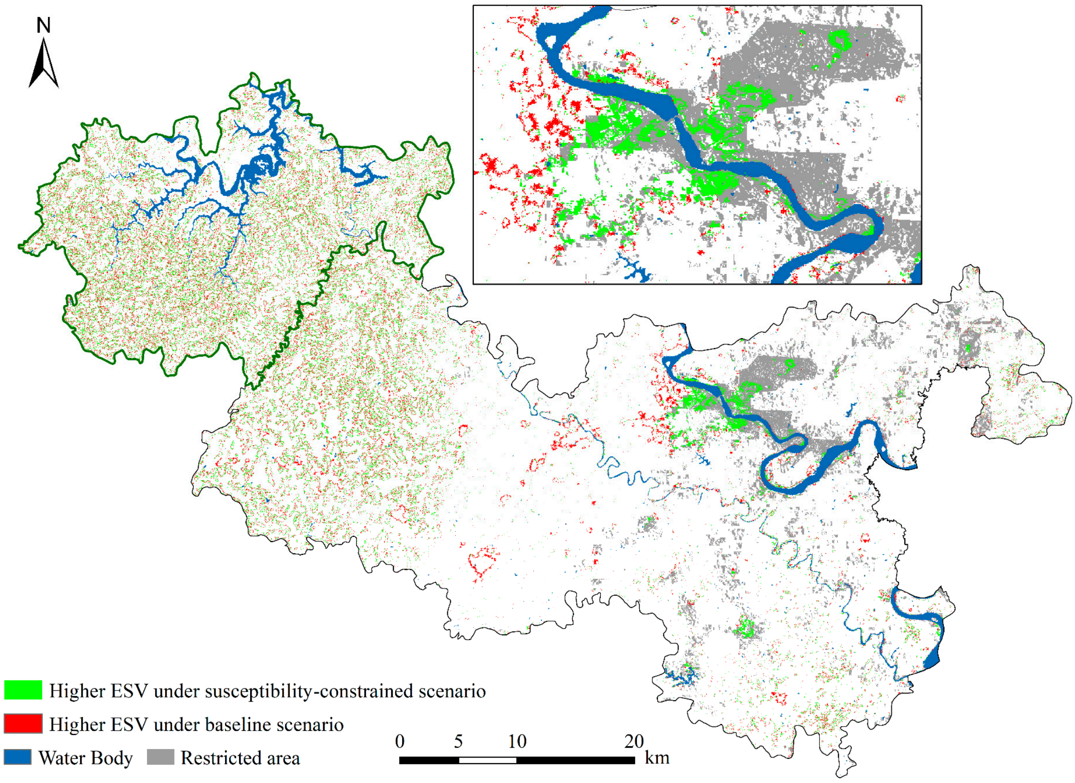

Figure S6. ESV gain–loss map of Nanbu County showing difference in ESV between the two 2050 scenarios (susceptibility-constrained minus baseline).

B. Supplementary tables

Table S1. Sown Area, Production, and Average Price in Nanbu County, 2020

Crops	Sown Area（hm²）	Production (tonnes)	Average Price (CNY/50kg)
Rice	19973	159687	130
Wheat	28793	169909	120
Corn	25120	106634	114

Table S2. Ecosystem service equivalent values per unit area for each land use type in Nanbu County

Ecosystem service functions		Arable Land	Forest Land	Grassland	Water Body
Provisioning services	Food production	1.003	0.220	0.380	0.800
	Raw material production	0.307	0.520	0.560	0.230
	Water supply	-0.775	0.270	0.310	8.290
Regulating services	Gas regulation	0.802	1.700	1.970	0.770
	Climate regulation	0.423	5.070	5.210	2.290
	Environmental purification	0.121	1.490	1.720	5.550
	Hydrological regulation	1.005	3.340	3.820	102.240
Supporting services	Soil conservation	0.724	2.060	2.400	0.930
	Nutrient cycling maintenance	0.141	0.160	0.180	0.070
	Biodiversity	0.154	1.880	2.180	2.550
Cultural services	Aesthetic landscape	0.069	0.820	0.960	1.890

Table S3. Ecosystem service value per unit area for each land use type in Nanbu County(CNY/hm²)

Ecosystem service functions		Arable Land	Forest Land	Grassland	Water Body
Provisioning services	Food oduction	2067.45	453.48	783.28	1649.02
	Raw material production	632.81	1071.86	1154.31	474.09
	Water supply	-1597.48	556.54	638.99	17087.93
Regulating services	Gas regulation	1653.14	3504.16	4060.70	1587.18
	Climate regulation	871.92	10450.64	10739.22	4720.31
	Environmental purification	249.41	3071.29	3545.38	11440.05
	Hydrological regulation	2071.58	6884.64	7874.05	210744.24
Supporting	Soil conservation	1492.36	4246.22	4947.05	1916.98

Ecosystem service functions		Arable Land	Forest Land	Grassland	Water Body
services	Nutrient cycling maintenance	290.64	329.80	371.03	144.29
	Biodiversity	317.44	3875.19	4493.57	5256.24
Cultural services	Aesthetic landscape	142.23	1690.24	1978.82	3895.80
Total		8191.50	36134.06	40586.40	258916.13

Table S4. The deterministic coefficients of each evaluation factors

Category	Number	Grade	Number of Landslide Points	Area in km²	PPa	CF
Elevation (m)	1	266~372	90	711.35	0.1265	0.029
	2	372~422	102	714.04	0.1428	0.156
	3	422~485	48	417.20	0.1151	-0.076
	4	485~566	24	245.03	0.0979	-0.228
	5	566~818	9	125.98	0.0714	-0.453
Slope (°)	1	0~5	35	395.74	0.0884	-0.31
	2	5~10	84	617.64	0.136	0.106
	3	10~20	112	864.49	0.1296	0.055
	4	20~30	40	288.91	0.1385	0.125
	5	>30	2	46.82	0.0427	-0.683
Aspect	1	Flat Land	1	14.18	0.0705	-0.461
	2	North	28	238.58	0.1174	-0.055
	3	Northeast	35	279.50	0.1252	0.017
	4	East	31	302.76	0.1024	-0.189
	5	Southeast	24	259.40	0.0925	-0.275
	6	South	32	259.22	0.1234	0.001
	7	Southwest	43	293.19	0.1467	0.181
	8	West	43	291.97	0.1473	0.185
	9	Northwest	36	274.80	0.131	0.067
Soil Type	1	Newly Deposited Soil	4	16.90	0.2367	0.546
	2	Purple Soil	204	1690.43	0.1207	-0.024
	3	Paddy Soil	49	372.03	0.1317	0.073
	4	Yellow Soil	13	55.79	0.233	0.537
	5	Lake	3	78.45	0.0382	-0.717
NDVI	1	<0.54	5	39.03	0.1281	0.043
	2	0.54~0.69	30	146.66	0.2046	0.453
	3	0.69~0.76	82	641.50	0.1278	0.04
	4	0.76~0.82	120	1149.68	0.1044	-0.172
	5	>0.82	36	236.73	0.1521	0.216
Surface Lithology	1	IB2/Basalt	10	39.20	0.2551	0.589

Category	Number	Grade	Number of Landslide Points	Area in km²	PPa	CF
	2	MA1/Quartzite	3	25.24	0.1189	-0.041
	3	SC2/Sand/Mixed sand/Feldspathic sandstone	227	1934.24	0.1174	-0.055
	4	SC2/SC4	3	36.89	0.0813	-0.371
	5	SC3/Silt/Mud/Mudstone	5	43.48	0.115	-0.076
	6	SC4/Shale	15	71.27	0.2105	0.472
	7	UF/Fluvial environment	3	16.43	0.1826	0.37
	8	UR1/Urban area	5	16.50	0.303	0.676
	9	WR/Lake	2	30.35	0.0659	-0.499
Annual Precipitation (mm)	1	983~1007	28	407.57	0.0687	-0.476
	2	1007~1028	41	544.32	0.0753	-0.421
	3	1028~1049	40	418.26	0.0956	-0.248
	4	1049~1066	98	479.26	0.2045	0.453
	5	1066~1088	66	364.19	0.1812	0.364
Annual Average Temperature (℃)	1	15.7~16.2	10	158.01	0.0633	-0.52
	2	16.2~16.5	31	342.10	0.0906	-0.292
	3	16.5~16.7	36	357.06	0.1008	-0.203
	4	16.7~17.0	128	848.19	0.1509	0.208
	5	17.0~17.4	68	508.24	0.1338	0.089
Distance to Road (m)	1	0~700	105	742.52	0.1414	0.146
	2	700~1500	79	611.80	0.1291	0.051
	3	1500~2500	45	495.69	0.0908	-0.29
	4	2500~3800	29	267.45	0.1084	-0.135
	5	>3800	15	96.14	0.156	0.239
Distance to Water System (m)	1	0~900	103	752.75	0.1368	0.113
	2	900~2000	75	609.15	0.1231	-0.002
	3	2000~3300	60	467.96	0.1282	0.043
	4	3300~4900	32	295.89	0.1081	-0.138
	5	>4900	3	87.85	0.0341	-0.749

Table S5. Logistic regression results for landslide susceptibility factors

Variable	Standard Error	Significance (p-value)	Regression Coefficient
Constant	0.077	<0.001	-1.094
Slope	0.493	0.002	1.518
Aspect	0.492	0.018	1.162
NDVI	0.39	0.002	1.198
Surface Lithology	0.433	0.005	1.216
Annual Precipitation	0.19	<0.001	1.586
Distance to Road	0.441	0.006	1.202
Distance to Water System	0.558	0.029	1.218

Table S6. Global Moran's I of the CF-LR residuals under alternative spatial-weight specifications

Spatial weight	Moran's I	z-score	*p* value
Inverse distance (1/d)	0.181	9.78	0.0001
Inverse distance squared (1/d²)	0.220	9.32	0.0001
Distance band (⩽ 2407 m)	0.137	8.43	0.0001
K-nearest neighbours (K = 8)	0.144	10.35	0.0001

Note: The weight matrices were all row-standardized. The thresholds for inverse distance and distance-band approaches were set to the maximum nearest-neighbor distance of 2407 m, ensuring that each point had at least one neighbor and that no isolated points remained. Z-scores were computed under the randomization assumption, and the corresponding *p*-values were derived from 9999 permutation tests (two-sided).

Table S7. Results of spatially blocked cross-validation for the CF-LR model

Validation scheme	Configuration	AUC
Blocked CV (regular grid)	3x3 = 9 blocks	0.665
Blocked CV (regular grid)	4x4 = 16 blocks	0.664
Blocked CV (regular grid)	5x5 = 25 blocks	0.671
Blocked CV (regular grid)	6x6 = 36 blocks	0.680
Blocked CV (k-means)	k=8 blocks	0.652 ± 0.004
Blocked CV (k-means)	k=10 blocks	0.657 ± 0.009
Blocked CV (k-means)	k=12 blocks	0.667 ± 0.008

Note: The modelling sample (N = 1092; 273 landslide, 819 non-landslide) was partitioned into spatially contiguous blocks using (i) regular grids of 9, 16, 25 and 36 cells and (ii) k-means clustering on the coordinates with k = 8, 10 and 12 (all block sizes larger than the residual semivariogram range of 2.2 km). A leave-one-block-out scheme was applied and the logistic regression was refitted on the training blocks and used to predict the held-out block. Predictions from all folds were pooled and the AUC were computed (Mann–Whitney). For k-means partitioning, clustering was repeated 10 times with different random initializations, and values are reported as mean ± SD across repetitions; regular-grid partitioning is deterministic and yields a single pooled AUC.

Table S8. One-factor-at-a-time sensitivity of the PLUS model in the model calibration stage

Iteration	Pgt	Ec	NS	Dw	PoS	FOM	ΔFOM
Base	**0.9**	**0.1**	**3**	**0.5**	**0.0001**	**0.0991**	/
1	**0.7**	0.1	3	0.5	0.0001	0.1004	1.3%
2	**0.5**	0.1	3	0.5	0.0001	0.0994	0.3%
3	**0.3**	0.1	3	0.5	0.0001	0.0987	-0.4%
4	**0.1**	0.1	3	0.5	0.0001	0.0992	0.1%
5	0.9	**0.3**	3	0.5	0.0001	0.0990	-0.1%
6	0.9	**0.5**	3	0.5	0.0001	0.0990	-0.1%
7	0.9	**0.7**	3	0.5	0.0001	0.0995	0.4%
8	0.9	**0.9**	3	0.5	0.0001	0.0989	-0.2%
9	0.9	0.1	**2**	0.5	0.0001	0.0991	0.0%

Iteration	Pgt	Ec	NS	Dw	PoS	FOM	ΔFOM
10	0.9	0.1	**4**	0.5	0.0001	0.0718	-27.5%
11	0.9	0.1	**5**	0.5	0.0001	0.0717	-27.6%
12	0.9	0.1	**6**	0.5	0.0001	0.0613	-38.1%
13	0.9	0.1	3	**0.1**	0.0001	0.0993	0.2%
14	0.9	0.1	3	**0.3**	0.0001	0.0992	0.1%
15	0.9	0.1	3	**0.7**	0.0001	0.0994	0.3%
16	0.9	0.1	3	**0.9**	0.0001	0.0990	-0.1%
17	0.9	0.1	3	0.5	**0.01**	0.0977	-1.4%
18	0.9	0.1	3	0.5	**0.001**	0.0989	-0.2%
19	0.9	0.1	3	0.5	**0.00001**	0.0994	0.3%
20	0.9	0.1	3	0.5	**0.000001**	0.0990	-0.1%

Note: FOM here denotes the Figure of Merit computed against susceptibility-constrained simulated 2020 map; ΔFOM was calculated as the rate of change in FOM for a given iteration relative to that obtained from the susceptibility-constrained 2020 simulation (i.e., the Base iteration). Pgt refers to the probability for random patch seeds, Ec refers to the decay coefficient, NS refers to the neighborhood range, PoS refers to the maximum proportion of random seeds, and Dw refers to the policy intensity.

Table S9. Long-term stability of the 2050 susceptibility-constrained simulation.

Scheme	Perturbation	Kappa
N1	Pgt = 0.5	0.8123
N2	Pgt = 1.0	0.8840
N3	NS = 2	0.9888
N4	Dw = 0.3	0.9885
N5	Dw = 0.7	0.9884
N6	PoS = 0.001	0.9855
N7	Pgt=0.7; NS=2; Dw=0.7; PoS=0.001	0.8412

Note: Kappa is computed between each perturbed 2050 simulation (N1–N7) and the susceptibility-constrained 2050 simulation reported in the main text. Pgt refers to the probability for random patch seeds, Ec refers to the decay coefficient, NS refers to the neighborhood range, PoS refers to the maximum proportion of random seeds, and Dw refers to the policy intensity. Pgt was set to 0.5 and 1.0, bracketing its Base value in Table S8. Ec was not tested in this experiment, as its variation across iterations in Table S8 had negligible effects on FOM. NS was fixed at 2, given that its effective range was ⩽3 (Table S8). Dw was tested at 0.3 and 0.7, again bracketing its Base value. PoS showed limited influence on FOM in Table S8; thus, only one alternative value (deviating from its Base) was examined.

Table S10. Land use transfer matrix under baseline scenario and susceptibility-constrained scenario (hm^2)

Land use type in 2020	Land use type in 2050					
	Arable Land	Forest Land	Grassland	Water Body	Construction Land	Total
**High-susceptibility zone under susceptibility-constrained scenario**						
Arable land	8445.41	103.73	1.40	25.30	54.07	8629.90
Forest land	62.94	135.71	0.00	0.22	0.00	198.88
Grassland	0.77	0.00	5.91	0.00	0.45	7.13
Water body	10.48	0.36	0.00	977.92	26.32	1015.08
Construction land	146.19	0.08	9.13	10.80	686.27	852.48
Total	8665.79	239.88	16.44	1014.25	767.11	10703.47
**Non-high-susceptibility zone under susceptibility-constrained scenario**						
Arable land	152775.74	18889.76	0.02	543.18	3452.46	175661.16
Forest land	3627.65	23557.51	0.00	9.38	2.37	27196.91
Grassland	0.64	0.06	0.19	0.00	6.43	7.33
Water body	189.32	18.27	0.00	5480.53	33.84	5721.96
Construction land	192.95	5.28	0.24	17.41	1906.13	2122.01
Total	156786.30	42470.89	0.45	6050.49	5401.23	210709.37
**High-susceptibility zone under baseline scenario**						
Arable land	7482.04	149.94	0.28	69.97	927.66	8629.88
Forest land	55.57	143.02	0.00	0.23	0.01	198.84
Grassland	0.18	0.00	0.73	0.00	6.19	7.10
Water body	5.80	0.38	0.00	977.26	31.63	1015.08
Construction land	35.63	0.08	0.67	12.96	803.10	852.45
Total	7579.21	293.42	1.68	1060.43	1768.59	10703.34
**Non-high-susceptibility zone under baseline scenario**						
Arable land	153833.32	18851.81	0.16	514.13	2461.63	175661.05
Forest land	3638.75	23547.74	0.00	8.80	1.72	27197.00
Grassland	0.91	0.00	0.24	0.00	6.19	7.33
Water body	189.35	19.21	0.00	5481.01	32.31	5721.89
Construction land	216.08	6.03	0.25	16.85	1882.78	2122.01
Total	157878.41	42424.79	0.65	6020.79	4384.63	210709.27

Table S11. Ecosystem service value disaggregated by land use type and susceptibility zone in Nanbu County (10^4 CNY)

**Year and scenario**	**Zones**	**Arable Land**	**Forest Land**	**Grassland**	**Water Body**	**Construction Land**	**Total**	**Incremental change**
Observed in 2020	Study area	150978.88	99015.09	58.69	174444.49	0	424497.15	-
	High-susceptibility zone	7069.65	718.74	28.94	26282.58	0	34099.91	-
	Non-high-susceptibility zone	143909.23	98296.35	29.75	148161.91	0	390397.24	-
Susceptibility-constrained scenario in 2050	Study area	135542.98	154368.53	68.55	182942.11	0	472922.17	48425.02
	High-susceptibility zone	7098.95	866.89	66.72	26261.6	0	34294.16	194.25
	Non-high-susceptibility zone	128444.03	153501.64	1.83	156680.51	0	438628.01	48230.77
Baseline scenario in 2050	Study area	135547.33	154402.68	9.46	183376.06	0	473335.53	48838.38
	High-susceptibility zone	6208.93	1060.5	6.82	27457.8	0	34734.05	634.14
	Non-high-susceptibility zone	129338.4	153342.18	2.64	155918.26	0	438601.48	48204.24
